# Targeting Hypoxic Tumors with Hybrid Nanobullets for Oxygen-Independent Synergistic Photothermal and Thermodynamic Therapy

**DOI:** 10.1007/s40820-021-00616-4

**Published:** 2021-03-31

**Authors:** Di Gao, Ting Chen, Shuojia Chen, Xuechun Ren, Yulong Han, Yiwei Li, Ying Wang, Xiaoqing Guo, Hao Wang, Xing Chen, Ming Guo, Yu Shrike Zhang, Guosong Hong, Xingcai Zhang, Zhongmin Tian, Zhe Yang

**Affiliations:** 1 The Key Laboratory of Biomedical Information Engineering of Ministry of Education, School of Life Science and Technology, Xi’an Jiaotong University, Xi’an, 710049 People’s Republic of China; 2 Paulson School of Engineering and Applied Sciences, John A, Harvard University, Cambridge, MA 02138 USA; 3 School of Engineering, Massachusetts Institute of Technology, Cambridge, MA 02139 USA; 4 School of Public Health, Guangxi Medical University, Nanning, 530000 People’s Republic of China; 5 Division of Engineering in Medicine, Department of Medicine, Brigham and Womens Hospital, Harvard Medical School, Cambridge, MA 02139 USA; 6 Department of Materials Science and Engineering, Stanford University, Stanford, CA 94305 USA

**Keywords:** Photothermal therapy (PTT), Thermodynamic therapy (TDT), Targeting hybrid nanobullet, Hypoxia tumor, Zinc phthalocyanine aggregate (ZPA)

## Abstract

Hypoxia is a feature of solid tumors and it hinders the therapeutic efficacy of oxygen-dependent cancer treatment. Herein, we have developed all-organic oxygen-independent hybrid nanobullets ZPA@HA-ACVA-AZ for the “precise strike” of hypoxic tumors through the dual-targeting effects from surface-modified hyaluronic acid (HA) and hypoxia-dependent factor carbonic anhydrase IX (CA IX)-inhibitor acetazolamide (AZ). The core of nanobullets is the special zinc (II) phthalocyanine aggregates (ZPA) which could heat the tumor tissues upon 808-nm laser irradiation for photothermal therapy (PTT), along with the alkyl chain-functionalized thermally decomposable radical initiator ACVA-HDA on the side chain of HA for providing oxygen-independent alkyl radicals for ablating hypoxic cancer cells by thermodynamic therapy (TDT). The results provide important evidence that the combination of reverse hypoxia hallmarks CA IX as targets for inhibition by AZ and synergistic PTT/TDT possess incomparable therapeutic advantages over traditional (reactive oxygen species (ROS)-mediated) cancer treatment for suppressing the growth of both hypoxic tumors and their metastasis.

**Supplementary Information:**

The online version contains supplementary material available at 10.1007/s40820-021-00616-4.

## Highlights

- An all-organic hybrid nanobullets labeled as ZPA@HA-ACVA-AZ NBs was developed for the “precise strike”of hypoxic tumors through an oxygen-independently synergistic PTT/TDT, possessing therapeutic advantages over traditional ROS-mediated cancer treatment.
- By feat of dual-targeting effect from surface-modified HA (targeting CD44 receptors) and AZ (targeting CA IX), the nanobullets accumulated at hypoxic tumors efficiently.
- The synergism of intelligent nanobullets could suppress the primary breast tumor growth and lung metastasis via CA IX inhibition by AZ and synergistic PTT/TDT.

**Supplementary Information:**

The online version contains supplementary material available at 10.1007/s40820-021-00616-4.

## Graphical abstract

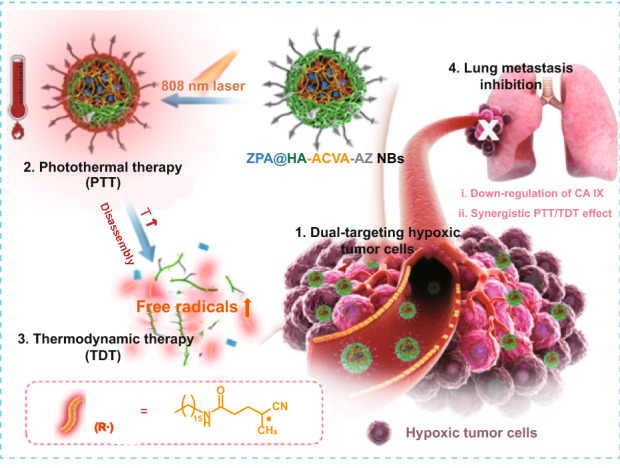

## Introduction

Phototherapies, including photodynamic therapy (PDT) and photothermal therapy (PTT), have shown great potential in cancer therapy due to their inherent high selectivity, noninvasive burden and superior controllability [1–4]. Besides, it has been reported that synergistic PDT/PTT can improve the limitations of each modality and achieve enhanced therapeutic outcomes [5–8]. However, the hypoxic tumor microenvironment (TME) sets up formidable barricades for combined PTT/PDT by inhibiting the generation of reactive oxygen species (ROS) during the oxygen-dependent PDT process [9–12]. Therefore, some other oxygen-independent strategies that maintain high level of cytotoxic free radicals under hypoxia condition have been explored to provide replacements for ROS-based cancer treatment. Among those strategies, thermodynamic therapy (TDT) becomes a new therapeutic modality during which the alkyl radicals can be generated efficiently upon heating by using thermally decomposable radical initiators as radical donors. Moreover, when combining TDT and PTT, the light-induced heat and heat-induced alkyl radicals can synergistically destroy cellular ingredients and induce cell death, ignoring tumor hypoxia [13–17]. However, the commonly selected radical initiator 2, 2′-azobis[2-(2-imidazolin-2-yl) propane] dihydrochloride (AIPH) has shown susceptibility to decomposition under the physiological condition that may cause unpredictable side effects to normal tissues. Moreover, it is difficult for hydrophilic AIPH to achieve high-level accumulation in the tumor, which nonetheless is essential to improve the effectiveness of treatment [15, 16]. Therefore, a strategy that can efficiently deliver both radical initiators and PTT agents to hypoxic tumors would be valuable for development of photonic thermodynamic cancer therapy.

There is an old saying that “adopt measures suiting local conditions.” Hypoxia, as a feature of solid tumors, hinders the therapeutic efficacy of oxygen-dependent cancer treatment. However, the hypoxic TME is also a double-edged sword since special hallmarks induced under hypoxia condition can be selected as targets to design the effective therapeutic strategy to break into the hypoxic niche and thus realize a selective ablation of hypoxic cells. For example, hypoxia is a key inducer of hypoxia-dependent factors such as carbonic anhydrase IX (CA IX), which is a transmembrane zinc metalloenzyme that acts as a catalyst of reversible hydration for carbon dioxide to bicarbonate ions and protons [18]. This cellular surface enzyme is known to be specifically overexpressed in tumors and linked to hypoxia through a strong transcriptional activation mediated via the hypoxia-inducible factor-1 (HIF-1) transcription factor [19, 20]. CA IX is also recognized as one of the important molecular events for the tumor cells with the acquisition of the metastatic phenotype [21]. Given that CA IX can potentially become an attractive target for hypoxic tumors and metastases hypoxic tumors, various CA IX inhibitors with high affinities to the extracellular domain of CA IX have been developed for highly selective and efficient inhibition of the catalytic activity of CA IX [22–24]. Moreover, there is an interesting perspective in the field of antitumor CA inhibitors, related to the development of nano-objects derivatized with CA IX inhibitors in recent years. Since the nanoparticles are totally membrane impermeable, which is a highly desirable feature for CA inhibitors to inhibit selectively only CA IX which possesses extracellular active site, the CA inhibitors (*e.g.,* acetazolamide, AZ) modified nanoplatforms could avoid the contact with intracellular CA isoforms and present high selectivity toward CA IX [25, 26]. By using the small molecule-based CA inhibitors as the moiety for tumor navigation, the therapeutic agents would target hypoxic tumor efficiently for enhancing cancer therapy and suppressing cancer metastasis simultaneously [27, 28].

**Fig. 1:**
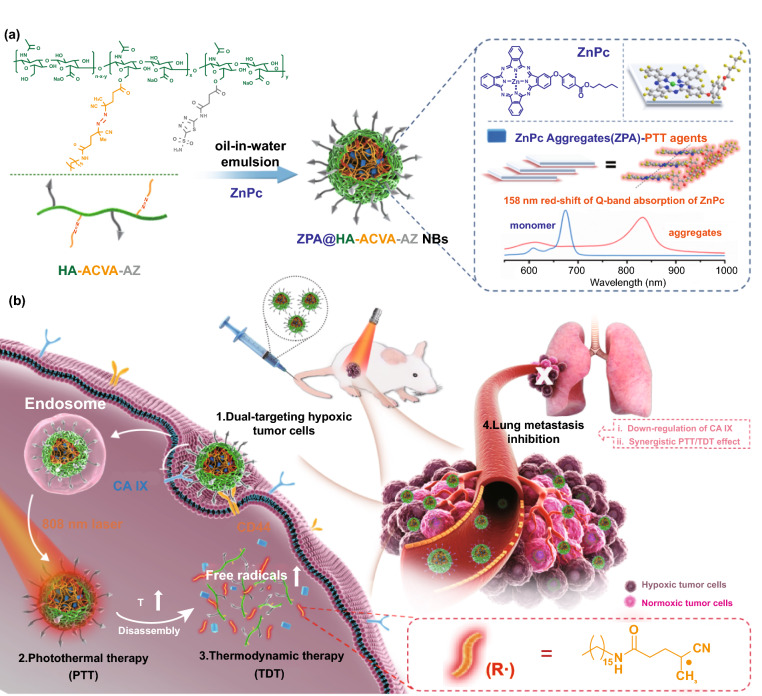
**a** Schematic illustration of the fabrication of ZPA@HA-ACVA-AZ NBs through oil-in-water emulsion method and formation of special ZnPc aggregates (ZPA) in the core of nanobullets. **b** Mechanistic action of ZPA@HA-ACVA-AZ NBs for synergistic photothermal thermodynamic therapy and lung metastasis-inhibition. The CD44/CA IX dual-targeting ZPA@HA-ACVA-AZ NBs display the enhanced accumulation in hypoxic tumors. Upon 808-nm laser irradiation, the light-induced heat and heat-induced alkyl radicals could synergistically induce cell death and suppress cancer metastasis via synergistic PTT/TDT and CA IX inhibition

To this end, the key issue is to develop useful strategies, beneficial from the tumor hypoxia, to kill hypoxic tumors via oxygen-irrelevant treatment. To achieve this goal, nanobullets (NBs), which are capable of loading various therapeutics as “gunpowder” and targeting lesions with surface-modified ligands as “navigators” and finally delivering drugs deep into diseased tissues, have great potential [29–36]. Herein, we developed all-organic nanobullets (denoted as ZPA@HA-ACVA-AZ NBs) to realize the “precise strike” of hypoxic tumors (Fig. 1). An amphiphilic hyaluronic acid (HA)-based lipoid (HA-ACVA-AZ) was prepared as the shell of nanobullets to provide dual-targeting effect for nanobullets toward hypoxic tumor cells overexpressing cluster determinant 44 (CD44) receptors and CA IX. Beyond, the loading pattern of radical initiators (ACVA) was optimized by conjugation of alkyl chain-functionalized initiators ACVA-HDA to HA, thus avoiding the premature release of radical initiators with undesirable side effect to normal tissues while enhancing the efficiency for targeted delivery of radical initiators to solid tumors. To better control the production of radicals upon laser irradiation, a special type of zinc(II) phthalocyanine aggregates (ZPA), as PTT agents, was formed in the inner cavity of nanobullets via an oil-in-water single emulsion method. Our previously published study demonstrated that ZPA displayed a large redshift of Q-band absorption from 674 nm (free ZnPc in DMSO) to 832 nm possibly due to the tilted side-by-side alignment of phthalocyanine molecules (J aggregates) with the exciton coupling effect of the transformed phthalocyanine aggregates [37]. 808-nm laser was thereby used to irradiate ZPA for efficient heat generation. Therefore, the sequentially generated heat and alkyl radicals could synergistically induce cell death and suppress cancer metastasis simultaneously via synergistic PTT/TDT and CA IX inhibition. Moreover, these all-organic nanobullets presented satisfactory biocompatibility, which could be employed as a powerful weapon to hit the hypoxic tumors via oxygen-independent PTT and photonic TDT.

## Experimental Section

### Materials, Cells and Animals

Hyaluronic acid (HA, Mw = 60 kDa) was obtained from Liuzhou Shengqiang Biotech Co. Ltd. (China). 4,4′-Azobis(4-cyanovaleric acid) (ACVA), acetazolamide (AZ), succinic anhydride, 1-hydroxybenzotriazole (HOBT), N,N-diisopropylethylamine (DIPEA), 4-dimethylaminopyridine (DMAP), tetrabutylammonium hydroxide (TBA-OH), coumarin 6 (C6), sodium chloride (NaCl), N-(3-dimethylaminopropyl)-N’-ethylcarbodiimide hydrochloride (EDC), ion exchange resin (Dowex 50wx8-400), palmitic acid (PA), dimethyl sulfoxide-*d*_*6*_ (DMSO-*d*_*6*_) and deuterium oxide (D_2_O) were obtained from Sigma-Aldrich (USA). Hexadecylamine (HDA) was purchased from Tokyo Chemical Industry (Japan). Hydrochloric acid (HCl) was purchased from International Laboratory USA (USA). 3-(4,5-Dimethyl-2-thiazolyl)-2,5-diphenyl-2H-tetrazolium bromide (MTT) was obtained from Sigma-Aldrich (USA). Hoechst 33,342 was purchased from ThermoFisher Scientific (USA). Tetrahydrofuran (THF), dichloromethane (DCM), methanol (MeOH), ethyl acetate (EA), sodium sulfate (Na_2_SO_4_), dimethyl sulfoxide (DMSO) and acetone were purchased from Tian Jin Fuyu Fine Chemical Co. Ltd. (China).

The 4T1 murine breast cancer cells ordered from American Type Culture Collection (ATCC) were cultured in Dulbecco’s modified Eagle medium (DMEM) supplemented with FBS (10%) and penicillin–streptomycin (100 units mL^−1^ and 100 μg mL^−1^, respectively). The cells in normoxic condition were grown at 37 ℃ in a humidified 5% CO_2_ atmosphere, and the cells in hypoxic condition were grown at 37 ℃ in a humidified 1% O_2_, 5% CO_2_ and 94% N_2_ atmosphere.

The protocol for animal experiments was approved by the Animal Experimentation Ethics Committee of Xi’an Jiaotong University. Female balb/c mice (5–6 weeks) were purchased and maintained in the Center for Experimental Animals at Xi’an Jiaotong University Health Science Center. The tumor volume was measured via a caliper. The length and width of each tumor were recorded to estimate the tumor volume according to the following formula:

1 $${\text{Volume}} = 0.5 \times {\text{length}} \times {\text{width}}^{2}$$

### Characterization

^1^H and ^13^C{^1^H} NMR spectra were recorded on a Bruker AVANCE III 600 spectrometer (^1^H, 400 MHz; ^13^C, 100.6 MHz). Spectra were referenced internally by using the residual solvent (^1^H, δ = 2.50 for DMSO-*d*_*6*_ and 4.79 for D_2_O for the most upfield signal) or solvent (^13^C, δ = 39.5 for DMSO-*d*_*6*_ for the most downfield signal) resonances relative to SiMe_4_. High-resolution mass spectra were recorded on a WATERS I-Class VION IMS QTOF and compared with the theoretical value. Dynamic light scattering (DLS) measurements were taken on a NanoBrook 90Plus (USA) for the determination of the particle size distribution and zeta potential (*ζ*). UV–Vis and fluorescence spectra were recorded on a Shimadzu UV-3600 UV–vis spectrophotometer and a Hitachi F7000 spectrofluorometer, respectively. Electron spin resonance (ESR) spectra were obtained from Bruker A300 Spectrometers (Germany).

### Synthesis of Amphiphilic Lipoid HA-ACVA-AZ

#### Synthesis of ACVA-HDA

A solution of ACVA (5.80 g, 20.69 mmol), EDC (0.87 g, 4.54 mmol), HOBT (0.62 g, 4.54 mmol), DIPEA (2 mL) in THF (150 mL) was stirred gently for 2 h on an ice bath in dark under a dry nitrogen atmosphere. After warming up to ambient temperature, HDA (1.00 g, 4.13 mmol) was added to the solution and stirred overnight under a nitrogen atmosphere at 25 °C. Then the solvent was removed under vacuum and the residue was re-dissolved in DCM. The extra HDA was removed by filtration, and ACVA was removed by repeated extraction by saturated NaHCO_3_. After the solvent was removed under vacuum, the residue was collected and purified using a silica gel column chromatography using DCM/MeOH (v/v, 20:1) to give a white solid (yield: 65%). ^1^H NMR (DMSO-*d*_*6*_): δ 0.85 (*t*, *J* = 4.4 Hz, 3H, CH_3_), 1.23–1.36 (*m*, 28H, CH_2_), 1.63–1.68 (*m*, 6H, CH_3_), 2.22–2.38 (*m*, 8H, CH_2_), 3.01 (*m*, 2H, CH_2_), 7.93 (*m*, 1H, NH), 12.42 (br, 1H, COOH). ^13^C{^1^H} NMR (DMSO-*d*_*6*_): δ 14.40, 22.53, 23.30, 23.40, 23.61, 26.85, 29.13, 29.17, 29.23, 29.42, 29.43, 29.47, 30.50, 31.72, 32.95, 33.68, 39.05, 72.01, 72.37, 118.64, 118.74, 170.10, 173.09 (some of the signals are overlapped). HRMS: m/z calcd for C_28_H_51_N_5_O_3_ [M + H]^+^, 504.3835, found 504.1390.

#### ***Synthesis of AZ-NH***_***2***_

AZ (3 g, 13.50 mmol) was dissolved in HCl (23.4 mL, 3 M) and refluxed for 3 h at 110 ℃. Then, NaOH (17.6 mL, 4 M) was added dropwise to the above solution on an ice bath followed by the extraction by EA (100 mL) for five times. Then, the organic phase was combined and dried by anhydrous Na_2_SO_4._ After filtration and solvent vacuum evaporation, the residues of AZ-NH_2_ were collected as white solid (yield: 74%). ^1^H NMR (DMSO-*d*_*6*_): δ 7.82 (*s*, 2H, NH_2_), 8.06 (*s*, 2H, NH_2_). ^13^C{^1^H} NMR (DMSO-*d*_*6*_): *δ* 158.49, 172.03. HRMS: *m*/*z* calcd for C_2_H_5_N_4_O_2_S_2_ [M + H]^+^, 180.9776, found 180.9840.

#### Synthesis of AZ-COOH

AZ-NH_2_ (0.45 g, 2.50 mmol) was reacted with succinic anhydride (0.275 g, 2.75 mmol) in 2.6 mL of DMF at 50 ℃ for 24 h. It was then precipitated by the addition of 30-fold volume of deionized water followed by the centrifugation (8000 rpm, 10 min). Then the supernatant was discarded and the pallet was further dried in vacuum to obtain a white solid (yield: 86%). ^1^H NMR (DMSO-*d*_*6*_): *δ* 2.60 (*t*, *J* = 6.9 Hz, 2H, CH_2_), 2.76 (*t*, *J* = 7.0 Hz, 2H, CH_2_), 8.36 (*s*, 1H, NH_2_), 12.61 (br, 1H, COOH). ^13^C{^1^H} NMR (DMSO-*d*_*6*_): δ 28.71, 30.42, 161.49, 164.71, 171.90, 173.79. HRMS: m/z calcd for C_6_H_8_N_4_O_5_S_2_Na [M + Na]^+^, 302.9936, found 302.9822.

#### Synthesis of HA-ACVA-AZ

Firstly, HA was converted to its tetrabutylammonium salt (HA-TBA) to improve the solubility of HA in DMSO. Briefly, the proton exchange resin was firstly rinsed by deionized water for three times via the centrifugation. Then HA (1 g) was dissolved in deionized water (50 mL) followed by adding resin (3 g) in the above solution for stirring at room temperature for 5 h, which allowed to exchange ions thoroughly. After removing the resin via filtration, the filtrate was titrated to a pH of 7.03 with TBA-OH. It was then subjected to lyophilization to give HA-TBA as a white solid.

Subsequently, a two-step esterification reaction was performed to prepare the HA-ACVA-AZ. Briefly, HA-TBA (0.6 g) was placed into a container with 25 mL of DMSO and stirred until dissolved completely. Simultaneously, ACVA-HDA (0.146 g, 0.29 mmol), EDC (0.112 g, 0.58 mmol) and DMAP (0.071 g, 0.58 mmol) were dissolved in another 5 mL of DMSO under gentle stirring at room temperature for 30 min. Then, two above solutions were mixed and stirred vigorously at room temperature in dark for 24 h. Then, a solution containing AZ-COOH (0.081 g, 0.29 mmol), EDC (0.11 g, 0.57 mmol) and DMAP (0.071 g, 0.57 mmol) pre-dissolved in 5 mL of DMSO for 30 min was added into the HA solution and stirred vigorously at room temperature for another 24 h. The mixture was then dialyzed against deionized water for 3 days (MW 3500 D) to remove DMSO. Subsequently, NaCl was added (1 g NaCl per 100 mL of solution) into the solution followed by precipitation into tenfold excess cold acetone. The precipitates were then collected via centrifugation and re-dissolved in deionized water for dialysis against water for 1 day. Finally, the amphiphilic lipoid HA-ACVA-AZ was obtained by lyophilization. The control amphiphilic lipoid HA-PA-AZ was prepared in similar methods by directly conjugating PA onto the side chain of HA instead of ACVA-HDA.

### Preparation of ZPA@HA-ACVA-AZ NBs

The lipoids emulsified hybrid nanobullets, including blank NBs (HA-ACVA-AZ NBs) and ZPA-loaded NBs (ZPA@HA-ACVA-AZ NBs), were prepared through an oil-in-water single emulsion method with the synthesized HA-ACVA-AZ as a surfactant. For the HA-ACVA-AZ NBs, 0.1 mL of DCM was added dropwise into 1 mL of HA-ACVA-AZ aqueous solution (5 mg mL^−1^) under stirring. The resulting emulsion was transferred into an ice bath equipped with a probe-type sonicator (Misonix Sonicator S-4000) while undergoing a probe sonication process (40% amplitude, 3 s power on, 3 s power off, 30 s). Subsequently, the organic phase was removed under slight vacuum condition to form the nanobullets along with the solvent evaporation. The obtained solution of nanobullets was centrifuged (4000 rpm, 10 min) to remove the large nanoparticles and unloaded components to give the purified HA-ACVA-AZ NBs. The ZPA-loaded ZPA@HA-ACVA-AZ NBs were fabricated by the same protocol, except that 0.1 mL of ZnPc in DCM/THF (v/v, 20:1) at concentration of 5 mg mL^−1^ was added into the initial HA-ACVA-AZ aqueous solution during the preparation.

The control nanobullets, including ZPA@HA-ACVA NBs and ZPA@HA-PA-AZ NBs, were formulated likewise by changing the aqueous solution HA-ACVA-AZ with the same volume of HA-ACVA solution and HA-PA-AZ solution at the same lipoid concentration.

Other nanobullets encapsulating the fluorescent dye coumarin 6 (C6) and IR780 iodide were also formed for the evaluation of cellular uptake, PTT effect and in vivo biodistribution, respectively. C6 (2.5 mg) or IR780 iodide (0.5 mg) was added into the corresponding lipoid solution to afford C6-loaded or IR780-loaded nanobullets.

### Evaluation of ZnPc Loading in Nanobullets

The amount of ZnPc loaded in nanobullets was investigated by recording the absorption spectra of ZPA-loaded nanobullets post-lyophilization in DMSO. The content of ZnPc in nanobullets could be determined according to the pre-established calibration curve of ZnPc in DMSO. The drug loading and entrapment efficiency of the nanobullets are determined based on Eqs. 2 and 3:

2 $${\text{Drug}}\;{\text{loading}}\;(\% ) = {{{\text{ZnPc}}\;{\text{amount}}\;{\text{in}}\;{\text{NBs}}} \mathord{\left/ {\vphantom {{{\text{ZnPc}}\;{\text{amount}}\;{\text{in}}\;{\text{NBs}}} {{\text{mass}}\;{\text{of}}\;{\text{NBs}}}}} \right. \kern-\nulldelimiterspace} {{\text{mass}}\;{\text{of}}\;{\text{NBs}}}} \times 100\%$$

3 $${\text{Entrapment}}\;{\text{efficiency}}\;(\% ) = {{{\text{Drug}}\;{\text{amount}}\;{\text{in}}\;{\text{NBs}}} \mathord{\left/ {\vphantom {{{\text{Drug}}\;{\text{amount}}\;{\text{in}}\;{\text{NBs}}} {{\text{drug}}\;{\text{feeding}}}}} \right. \kern-\nulldelimiterspace} {{\text{drug}}\;{\text{feeding}}}} \times 100\%$$

### Study of Stability of Nanobullets

The stability of HA-ACVA-AZ NBs and ZPA@HA-ACVA-AZ NBs in serum was determined by measuring their changes in size and polydispersity index (PDI) over a period of time. Briefly, the nanobullets were added into the solution of phosphate-buffered saline (PBS)/fetal bovine serum (FBS) (10%, v/v) and kept at 37 ℃ under gentle shaking. Then, 50 μL of sample was withdrawn and diluted for DLS measurement at predetermined time points. Besides, the nanobullets were also added into the PBS at various pH values, including pH 7.4, 6.8 and 5.5, followed by incubating at 37 ℃ under gentle shaking. Then, 50 μL of sample was withdrawn and diluted for DLS measurement at predetermined time points.

As for evaluating the photostability of nanobullets, the sample suspended in PBS (pH 7.4) were incubated at 37 ℃ under gentle stirring. 200μL of sample was taken out for absorption measurement at predetermined time points. The photostability of IR780@HA-ACVA-AZ NBs was also evaluated as control at an IR780 concentration of 20 μM.

### Investigation of Thermal Responsive Properties of Nanobullets

Blank HA-ACVA-AZ NBs dispersed in PBS (pH 7.4) were kept at the temperature of 25 ℃ and 60 ℃ for 24 h under gentle stirring. Then, 50 μL of sample was withdrawn and diluted for DLS measurement at preset time points. Additionally, the blank HA-ACVA-AZ NBs and ZPA@HA-ACVA-AZ NBs suspended in PBS (pH 7.4) were illuminated with a laser (808 nm, 1 W cm^−2^, 20 min) and the size of nanobullets was recorded to compare with ones without laser irradiation.

### Evaluation of Photothermal Effect of ZPA@HA-ACVA-AZ NBs

The ZPA@HA-ACVA-AZ NB solution ([ZnPc] = 10–40 μM, 3 mL) was placed into a 1-cm path length quartz cuvette. Then it was irradiated by a fiber-coupled continuous semiconductor diode laser (808 nm, 0.5–2 W cm^−2^, 10 min). Three other samples, including ZnPc dissolved in DMSO, ZnPc dissolved in DMSO/H_2_O (v/v, 1:20) and water, were studied for control. The position of laser was adjusted to cover the entire sample surface. Every 10 s, the solution temperature was monitored and recorded by a thermal couple probe (UT320D, UNI-T, China).

### Evaluation of Photothermal Stability of ZPA@HA-ACVA-AZ NBs

In order to study the photothermal stability of ZPA@HA-ACVA-AZ NBs, NB solution ([ZnPc] = 20 μM, 2.5 mL) was added into a 1-cm path length quartz cuvette. Then it was irradiated by a fiber-coupled continuous semiconductor diode laser (808 nm, 3 W cm^−2^, 6 min). Then, the laser was turned off and the temperature of solution was monitored continuously for 18 min until cooled to the room temperature. Additionally, every 10 s the temperature was recorded using a digital thermometer and the same measurement was repeated for another two cycles. For comparison, the IR780@HA-ACVA-AZ NBs was examined under the same treatment.

### Determination of Photothermal Conversion Efficiency of ZPA@HA-ACVA-AZ NBs

The ZPA@HA-ACVA-AZ NBs ([ZnPc] = 20 μM, 3 mL) was added into a 1-cm path length quartz cuvette. Then, it was irradiated with continuous laser (808 nm) for 21 min until temperature unchanged. Then the laser was turned off and the temperature of suspension was continuously monitored and recorded over a period of 40 min. Every 10 s, the temperature was recorded using a digital thermometer. As a control group, deionized water was examined under the same treatment. According to a reported method, the photothermal conversion efficiency (η) is calculated by Eq. 4:

4 $$\eta = \frac{{hS\left( {T_{{{\text{max,sample}}}} - T_{{{\text{surr}}}} } \right) - Q_{{{\text{dis}}}} }}{{I(1 - 10^{{ - A_{\lambda } }} )}}$$

In the above formula, *h* represents heat transfer coefficient, *S* represents the surface area of the quartz cuvette, *T*_surr_ is the surrounding temperature, and *T*_max, sample_ is the final temperature of the sample solution. In the denominator, *I* represents the incident laser power in *W* and A_λ_ represents the sample absorbance (808 nm). *Q*_dis_ is the energy imputed by the deionized water system which can be calculated by Eq. 5:

5 $$Q_{{{\text{dis}}}} = hS(T_{{{\text{max}}.{\text{H}}_{{2}} {\text{O}}}} - T_{{{\text{surr}}}} )$$

Through the following formula, the dimensionless temperature driving force *hS* can be calculated by Eq. 6:

6 $$hS = \frac{{\sum {m_{i} c_{i} } }}{\tau }$$

where *m* and *C* represent the mass (3 g) and heat capacity (4.2 J g^−1^) of water, respectively. And τ represents the sample system time constant which can be calculated by Eq. 7:

7 $$\tau = - \frac{t}{\ln (\theta )}$$

where *θ* is the dimensionless driving force and *t* represents time, which can be calculated based on Eq. 8:

8 $$\theta = \frac{{T - T_{{{\text{surr}}}} }}{{T_{{{\text{max}}}} - T_{{{\text{surr}}}} }}$$

where *T* represents the sample solution temperature after removing the laser at a predetermined time point. In this work, τ is equal to 363.25, m is 3 g and C is 4.2 J g^−1^, hS was calculated to be 0.0347 [Eq. (6)]. Through substituting *T*_max, H2O_ = 19.5 °C and *T*_surr_ = 17.7 °C into Eq. (5), *Q*_dis_ = 0.06246 J. By substituting *I* = 1 W, A_808_ = 0.545, *T*_max_ = 33.2 °C and *T*_surr_ = 19.4 °C into Eq. (4), the photothermal conversion efficiency was calculated to be 58%.

### Hemolysis and Erythrocyte Agglutination Assay

For evaluating the hemocompatibility of blank and ZPA loaded HA-ACVA-AZ NBs, erythrocytes were gathered and incubated with nanobullets at varying concentrations (50–300 μg mL^−1^) at the normal body temperature (37 ℃) for a day. Triton X-100 (1%, v/v) and PBS (pH 7.4, 0.01 M) solution were used as positive and negative controls, respectively. The hemolysis phenomenon induced by HA-ACVA-AZ NBs or control groups after incubation was determined by measuring the concentration of released hemoglobin collected by centrifuging (2000 rpm, 5 min). The hemolytic activity (%) of different groups is investigated by Eq. 9:

9 $$Hemolysis\;(\% ) = {{(A_{{{\text{sample}}}} - A_{{{\text{PBS}})}}} \mathord{\left/ {\vphantom {{(A{\text{sample}} - A{\text{PBS}})} {(A{\text{Triton}} - A{\text{PBS}}}}} \right. \kern-\nulldelimiterspace} {(A_{{{\text{Triton}}}} - A_{{{\text{PBS}}}}}}) \times 100\%$$

where *A*_Sample_, *A*_PBS_ and *A*_Triton_ represent the absorbance intensity of hemoglobin (410 nm) in HA-ACVA-AZ NBs, PBS and Triton X-100 groups, respectively. Then, the agglutination of erythrocyte induced by ZPA@HA-ACVA-AZ NBs and control samples were observed under a microscope.

### Detection of ABTS^+·^ Free Radicals

By taking advantage of the interaction between ABTS and toxic free radicals generated by free radical initiators ACVA-HDA, the production of ABTS^+•^ was executed. The mixture solution of ABTS (2 mg mL^−1^, 0.5 mL) and HA-ACVA-AZ NBs (5 mg mL^−1^, 0.5 mL) or HA-PA-AZ NBs (5 mg mL^−1^, 0.5 mL) was incubated at 50 °C for 2 h. At preset time points, an aliquot of sample (200 μL) was taken out for measuring their UV–vis spectra ranging from 450 and 900 nm. Then, the HA-ACVA-AZ NBs were mixed with ABTS followed by incubation at various temperatures for 2 h. Each aliquot of sample (200 μL) was taken out for the absorbance measurement at preset time points.

The production of ABTS^+•^ under 808-nm laser illumination was measured by the similar protocol. Briefly, the ZPA-loaded nanobullets, including ZPA@HA-ACVA-AZ NBs and ZPA@HA-PA-AZ NBs ([NBs] = 5 mg mL^−1^), were mixed with ABTS (2 mg mL^−1^, 0.5 mL) followed by laser irradiation (808 nm, 1 W cm^−2^, 10 min). The increment of solution absorbance at 736 nm was recorded at preset time points (0, 2, 4, 6, 8, 10, 12 min).

### Cellular Uptake of C6-loaded Nanobullets in vitro

About 2 × 10^5^ 4T1 cells in DMEM (2 mL) were seeded on each well of a 6-well plate and incubated at the temperature of 37 °C in a humidified 5% CO_2_ atmosphere overnight. Then the cells were further cultured in normoxic or hypoxic condition for another 24 h. Subsequently, after removing medium, the C6-loaded NBs (C6@HA-ACVA NBs and C6@HA-ACVA-AZ NBs, [C6] = 2 μM, 2 mL) were added into each well. After 4-h incubation in normoxic condition or hypoxic condition, the cells were rinsed twice by PBS, harvested by 0.25% trypsin–EDTA and then centrifuged. The cell pellet was also rinsed with PBS and subjected to another centrifugation. After resuspending the cells into cold PBS (0.5 mL), the fluorescence signal of cells was monitored and analyzed by a flow cytometer (Becton Dickinson, San Jose) with 10^4^ cells in each sample. The C6 were excited by a laser of 488 nm. Further, the cells were pre-incubated with HA (10 mg mL^−1^) or AZ (0.1 mg mL^−1^) dissolved in a FBS-free culture medium (2 mL) for 2 h at 37 °C for competitive inhibition experiment. The C6-loaded nanobullets in medium containing HA (10 mg mL^−1^) or AZ (0.1 mg mL^−1^) were then placed into each well for cell incubation at 37 °C for another 4 h. The cells were finally collected for flow cytometric analysis as the same method described above.

The cellular uptake of C6-loaded NBs was also examined by confocal laser scanning microscopy (CLSM). Approximately 1 × 10^5^ 4T1 cells in DMEM (2 mL) were seeded on a confocal dish and incubated at 37 °C in a humidified 5% CO_2_ atmosphere overnight. Then the cells were further cultured in normoxic or hypoxic condition for another 24 h. The similar treatment as the flow cytometric analysis was performed. After the incubation of cells and nanobullets, the medium was removed. Then the cells were washed twice by cold PBS. After using Hoechst 33,342 (10 μg mL^−1^, 1 mL) to stain the cell nuclei for 30 min, the intracellular distribution of nanobullets was visualized directly by CLSM (Leica TCS SP8 STED 3X). The Hoechst 33,342 and C6 were excited by 405 nm and 488 nm, respectively. The emission was monitored ranging from 410 to 498 nm and 493 to 635 nm, respectively.

### Intracellular Free Radical Generation

The intracellular free radical generation was detected by utilizing 2′,7′-dichlorofluorescein diacetate (DCFH-DA) as a chemical probe. Typically, approximately 1 × 10^4^ 4T1 cells, pre-incubated in normoxic or hypoxic condition for 24 h, were incubated with ZPA@HA-ACVA-AZ NBs, ZPA@HA-PA-AZ NBs or ZPA@HA-ACVA NBs at various concentrations ([ZnPc] = 2.5–40 μM) for 4 h. Then, the non-internalized NBs were rinsed twice by PBS. The DCFH-DA in PBS (100 μL, 10 μM) was subsequently placed into each well and incubated for 30 min. Then, the cells were rinsed entirely and refilled with 100 μL of PBS before being irradiated by a laser (808 nm, 1 W cm^−2^, 10 min). The fluorescence intensity in each well was measured by a BioTek microplate reader with an excitation filter of 495 nm and an emission filter of 525 nm.

As control, free ZnPc was also incubated with 4T1 cells for 4 h. The cells were then rinsed twice by PBS and DCFH-DA was subsequently placed into each well and incubated for 30 min. Then, the cells were rinsed entirely and refilled with 100 μL of PBS before being irradiated by a laser (635 nm, 30 mW cm^−2^, 5 min). Finally, the fluorescence intensity in each well was determined as mentioned above.

### Cytotoxicity Assay

To survey the cytotoxicity of ZPA@HA-ACVA-AZ NBs, MTT assay was applied. Briefly, about 1 × 10^4^ 4T1 cells in DMEM (2 mL) were seeded on each well of a 96-well plate and incubated at temperature of 37 °C in a humidified 5% CO_2_ atmosphere for 12 h. Then the cells were further cultured in normoxic or hypoxic condition for another day. Then, the culture medium involving various concentrations of nanobullets ([NBs] = 12.5–400 μg mL^−1^ for blank nanobullets, [ZnPc] = 1.25–40 μM for ZPA-loaded nanobullets) was added. The cells were cultured with nanobullets for 4 h. After the cells was washed with PBS for twice, the cells were exposed to a laser for 10 min (808 nm, 1 W cm^−2^) if needed. Afterward, the cells were further cultured for 24 h. For the MTT assay, 20 μL of MTT in PBS (5 mg mL^−1^) was added into each well, and cells were treated for another 4 h. Finally, after removing the culture medium gently, 150 μL of DMSO was added, and absorbance of each well was inspected. The cell viability was then determined by Eq. 10:

10 $$\% {\text{viability}} = \left[\sum\left(A_{i}/{A_{control}}\times100\right)\right]/{n}$$

where *A*_i_ represents the absorbance of the *i*_th_ data (*i* = 1, 2, …, n), *A*_control_ represents the average absorbance of the control wells in which no formulation was treated and n represents the number of the data points.

As control, free ZnPc-mediated PDT efficacy was also investigated by adding free ZnPc into 4T1 cells, pre-incubated in normoxic or hypoxic condition, for 4 h. The cells were then rinsed twice by PBS before being irradiated by a laser (635 nm, 30 mW cm^−2^, 5 min). Afterward, the cells were further cultured for 24 h. Finally, the cytotoxicity was determined as mentioned above.

### Inhibition of Cell Migration

Wound-healing assay and transwell assay were used for measuring the inhibitory effect of ZPA@HA-ACVA-AZ NBs on 4T1 cell migration. 4T1 cells at a density of 3 × 10^5^ cells/well were seeded in a 24-well plate and incubated in hypoxic condition for wound-healing assay. Then, a sterile pipette was used to draw the lines in each well. The culture medium was further replaced by a fresh medium containing different drug formulations. Following 4 h of treatment, the cells were rinsed by PBS and subjected to 808-nm laser irradiation for 10 min (1 W cm^−2^). Then the cells were cultured in hypoxic condition for another 20 h and migration distance was measured using an inverted microscope (Olympus IX53).

4T1 cells were seeded in serum-free medium containing different drug formulations in the apical chamber for transwell assay. Subsequently, the apical chambers were placed in basolateral chambers with the medium containing 50% FBS. After 4 h of incubation, the apical chamber was subjected to a laser (808 nm, 1 W cm^−2^, 10 min) followed by incubation for further 20 h. In the apical chamber, the non-migrated cells were then removed. The migrated cells were stained with crystal violet (0.1%) and photographed under an inverted microscope. Finally, the stained cells were decolorized with acetic acid (30%), and the absorbance of decolorization solution (540 nm) was measured using a microplate reader (Tecan M200) to estimate the migration rate quantitatively.

### Western Blot Analysis of CA IX in Different Cells under Different Treatments

Approximately 1 × 10^6^ 4T1 cells in DMEM (2 mL) were cultured on dishes of 10 cm diameter and incubated at the temperature of 37 °C in a humidified 5% CO_2_ atmosphere overnight. The cells were then cultured in normoxic or hypoxic condition for another 24 h. Subsequently, the medium was removed and nanobullets including HA-ACVA-AZ NBs, ZPA@HA-ACVA NBs and ZPA@HA-ACVA-AZ NBs ([ZnPc = 10 μM]) were subsequently added into each well. After 4-h incubation in normoxic or hypoxic condition, the cells were collected by trypsin–EDTA (0.25%, 0.5 mL, Invitrogen) for 5 min and centrifuged. The cell pellet was rinsed with fresh medium and subjected to laser irradiation (808 nm, 1 W cm^−2^, 10 min) if needed, followed by reseeded onto the 10 cm culture dishes for a further 24-h incubation in normoxic condition or hypoxic condition. For the Western blot analysis, the cells were lysed using RIPA containing protease inhibitors and PMSF inhibitors. Equal amounts of proteins were electrophoresed on polyacrylamide gels (12%) and transferred to a polyvinylidene fluoride membrane. After blocking with skim milk powder (5%, 1 h), primary antibodies (mouse anti-*β*-actin(8H10D10), 1:1000, Cell Signaling Technology, P60709; rabbit anti-CA9, 1:1000, proteintech,11,071–1-AP) were bound at the temperature of 4 °C overnight and then washed by tris-buffered saline-tween20 (TBS T). Further, membranes were probed with horseradish peroxidase (HRP)- conjugated antirabbit IgG (1:3000, CWBIO, CW0103S) antibodies for 1 h. Protein bands were visualized using enhanced chemiluminescence (ECL) by ChemiScope 3300 mini.

### Biodistribution of Nanobullets in Tumor-bearing Mice

To examine the biodistribution of nanobullets in 4T1 tumor-bearing mice, the near-infrared fluorescent dye, IR780, was encapsulated into HA-ACVA NBs and HA-ACVA-AZ NBs. Until the volume of the subcutaneous 4T1 tumor reached 200–300 mm^3^, mice were randomly divided into three groups. Free IR780, IR780@HA-ACVA NBs and IR780@HA-ACVA-AZ NBs were intravenously administrated into mice, respectively. (The dosage of IR780 was calculated as 0.3 mg per kg mouse.) At preset intervals (2, 4, 7, 10 and 24 h), the fluorescent images were captured using in vivo imaging system (IVISs Lumina XR Series III, PerkinElmer). Finally, the mice were sacrificed and major tissues (heart, liver, spleen, lung, kidney and tumor) were harvested for ex vivo imaging.

### Pharmacokinetic Studies

Free ZnPc and ZPA@HA-ACVA-AZ NBs were *i.v.* injected into the nude balb/c mice at ZnPc doses of 2 mg per kg mouse (*n* = 5), respectively. At predetermined time points (0, 1, 6, 12 and 24 h), the blood samples were collected via eye puncture. Then the blood was centrifuged at 4 °C (2500 g, 20 min) and the supernatant plasma was collected. After lyophilization, the residue of plasma was dissolved in DMSO to extract the ZnPc and subjected to fluorescence measurement. (*E*_ex_ = 630 nm, *E*_em_ = 685 nm) to calculate the concentration of ZnPc.

### In vivo Thermal Imaging

When the volume of the subcutaneous 4T1 tumor reached 200–300 mm^3^, mice were randomly divided into six groups. 0.9% NaCl, free ZnPc in DMSO/PBS (v/v, 1/20), HA-ACVA-AZ NBs, ZPA@HA-ACVA NBs and ZPA@HA-ACVA-AZ NBs were intravenously administrated into mice, respectively (200 μL, the dosage of ZnPc was calculated as 0.8 mg per kg mouse for ZPA-loaded nanobullets and the dosage of nanobullets was calculated as 10 mg per kg mouse for blank nanobullets). After 24 h, the mice were irradiated by a laser (808 nm, 1 W cm^−2^, 6 min) and the temperature at tumor region was recorded with an infrared thermographic camera (Fluke TiS20, USA).

### In vivo Therapeutic Efficacy

In vivo therapeutic efficiency of ZPA@HA-ACVA-AZ NBs was investigated in balb/c mice bearing subcutaneous 4T1 tumors. Until the tumor volume reached to 100–150 mm^3^, the mice were randomly divided into five groups (*n* = 10) and intravenously administrated NaCl (0.9%) and different drug formulations at the same ZnPc doses (0.8 mg per kg mouse). Then, the tumor tissues of experimental group were irradiated with a laser (808 nm, 1 W cm^−2^, 10 min) at 24 h post-injection. The tumor volumes and body weights were measured and recorded daily during the experiment. On day 7, five mice from each group were sacrificed and their tumors were then collected and stained with TUNEL assay and H&E, respectively.

### In vivo Biosafety

For studying the biosafety of ZPA@HA-ACVA-AZ NBs, balb/c mice received the same treatments as mentioned above and the organs (heart, liver, spleen, kidney) of mice were collected for H&E staining after 21 days. Besides, blood samples were harvested by retro-orbital bleed for routine blood examination and centrifuged (1800 g, 15 min, 4 °C) for the plasma. Further, the content of aspartate transaminase (AST), creatinine (CRE), alanine aminotransferase (ALT) and urea nitrogen (BUN) in plasma was also measured.

### Statistical Analysis

All experimental data were presented as the mean values ± standard deviations (SD), which were also analyzed through Student’s *t*-test. P* < 0.05 or P** < 0.01 was to be statistically significant and extremely significant, respectively.

## Results and Discussion

### Preparation and Characterization of ZPA@HA-ACVA-AZ NBs

**Fig. 2:**
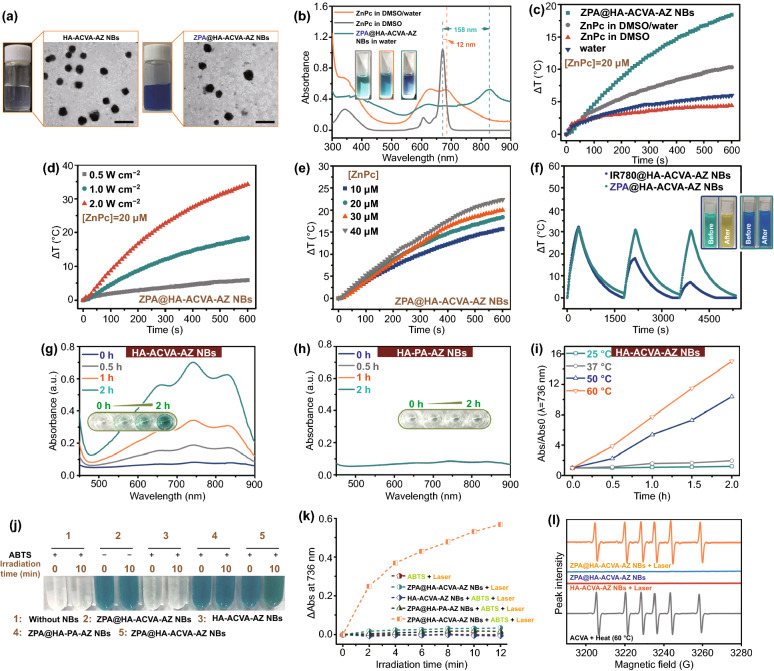
Characterizations of ZPA@HA-ACVA-AZ NBs.** a** Digital images and TEM images of blank HA-ACVA-AZ NBs and ZPA@HA-ACVA-AZ NBs (scale bars: 500 nm). **b** Electronic absorption spectra of ZPA@HA-ACVA-AZ NBs in deionized water, ZnPc in DMSO/water (5/95, v/v) and ZnPc in DMSO and digital images of corresponding solution (insets). **c** Temperature variation of various samples as a function of the irradiation time at a power density of 1 W cm^−2^ ([ZnPc] = 20 μM for ZPA@HA-ACVA-AZ NBs, ZnPc in DMSO/water and ZnPc in DMSO). **d** Plots of the temperature variation of ZPA@HA-ACVA-AZ NBs over 10 min upon irradiation at different laser power densities ([ZnPc] = 20 μM). **e** Plots of the temperature increase of ZPA@HA-ACVA-AZ NBs over 10 min upon irradiation at different concentrations of ZPA@HA-ACVA-AZ NBs at a power density of 1 W cm^−2^. **f** Temperature curves for ZPA@HA-ACVA-AZ NBs ([ZnPc] = 20 μM) and IR780@HA-ACVA-AZ NBs ([IR780] = 20 μM) upon 808-nm laser irradiation (3 W cm^−2^) repeatedly (insets: digital images of NB solution before or after laser irradiation). **g** Generation of ABTS^+•^ as induced by the free radicals released from blank HA-ACVA-AZ NBs at 50 ℃ with different incubation times. **h** Generation of ABTS^+•^ as induced by the free radicals released from blank HA-PA-AZ NBs at 50 ℃ with different incubation times. **i** Absorbance of ABTS^+•^ at 736 nm generated from the reaction of ABTS and blank HA-ACVA-AZ NBs under 0–2-h incubation at various temperatures.** j** Images of various samples with or without laser irradiation (1 W/cm^2^, 10 min) in the presence of ABTS. **k** Absorbance of ABTS^+•^ at 736 nm generated from the reaction of ABTS and ZPA@HA-ACVA-AZ NBs under 0–12 min laser irradiation. **l** Electron spin resonance (ESR) spectrum of DMPO in ZPA@HA-ACVA-AZ NBs with or without irradiation (ZPA@HA-PA-AZ NBs with irradiation and ACVA with heat treatment as control)

ACVA-HDA and AZ-COOH were first synthesized (Fig. S1a, b) and characterized through nuclear magnetic resonance (NMR) spectra (Figs. S2-S4) and high-resolution mass spectra (Fig. S5). Then, three batches of lipoids HA-ACVA-AZ-***1–3*** (with different grafting ratios of ACVA-HDA) were prepared via a two-step esterification reaction (Fig. S1c). The grafting degrees of ACVA-HDA and AZ-COOH, calculated by ^1^H NMR analysis (Fig. S6), are summarized in Table S1. Another lipoid HA-PA-AZ without radical initiator was also synthesized as control (Fig. S7). Subsequently, the HA-ACVA-AZ NBs-***1–3*** and ZPA-loaded NBs (termed as ZPA@HA-ACVA-AZ NBs-***1–3***) were formulated via the oil-in-water emulsion method. As shown in Table S2, the size of ZPA-loaded nanobullets was around 300 nm and displayed the negative ζ-potential of about − 30 mV owing to the presence of anionic HA on their surface. Moreover, ZPA@HA-ACVA-AZ NBs-***2*** prepared from lipoid HA-ACVA-AZ-***2*** with grafting degree of 8.1% for ACVA-HDA and 18.8% for AZ presented the optimal ZPA entrapment efficiency (89.65%) and loading content (8.23%), demonstrating superior properties of this HA-ACVA-AZ in the formation of drug delivery nanosystems. Hence, ZPA@HA-ACVA-AZ NBs-***2*** was selected for all subsequent experiments. The nearly spherical shape of nanobullets was further observed through transmission electron microscopy (TEM) (Fig. 2a), and the size of nanobullets remained unchanged in a simulated physiological condition for 48 h, indicating their good serum stability (Fig. S8a). We also evaluated the stability of ZPA@HA-ACVA-AZ NBs in PBS at various pH values, including pH 7.4, 6.8 and 5.5. Based on the results shown in Fig. S8b, there did not appear a significant change on the size of nanobullets incubating at 37 °C in PBS of all pH values for 48 h. These results further verified the good stability of ZPA@HA-ACVA-AZ NBs.

As we expected, ZPA@HA-ACVA-AZ NBs exhibited a large redshift of 158 nm for ZnPc’s Q-band absorption (from 674 to 832 nm) upon the formation of ZPA in nanobullets (Fig. 2b). From the insets, the color of ZPA@HA-ACVA-AZ NBs was dark blue, which was different from other groups. Furthermore, ZPA@HA-ACVA-AZ NBs displayed no fluorescence emission (Fig. S9) and could not generate ^1^O_2_ upon 808-nm laser irradiation (Fig. S10). Since fluorescence and intersystem crossing for phototherapeutic agents compete with heat generation, the quenching of both fluorescence and ROS suggested that ZPA@HA-ACVA-AZ NBs could serve as promising PTT agents.

### Photothermal Properties and Free Radical Effect of ZPA@HA-ACVA-AZ NBs

To evaluate the photothermal properties of ZPA@HA-ACVA-AZ NBs, the sample was exposed to 808-nm laser irradiation continuously. After irradiation for 10 min, the temperature of ZPA@HA-ACVA-AZ NBs increased by 18.4 °C and this increment was much larger than other samples (Fig. 2c). Besides, the temperature increment of ZPA@HA-ACVA-AZ NBs increased with the growth of laser power density (Fig. 2d) and their concentration (Fig. 2e). Additionally, ZPA@HA-ACVA-AZ NBs presented promising photostability (Fig. S11) and photothermal stability (Fig. 2f), conquering the disadvantage of commercial PTT agents (*e.g.,* IR780) of rapid degradation in an aqueous solution or upon laser irradiation. The photothermal conversion efficiency of ZPA@HA-ACVA-AZ NBs was further calculated to be 58% (Fig. S12), which was higher than that of most reported PTT agents [38–41].

Alkyl chain-functionalized radical initiators ACVA-HDA in lipoids was designed to generate alkyl radicals upon heat treatment. Firstly, the radical generation of ACVA-HDA was observed in DMF by using 1,3-diphenylisobenzofuran (DPBF) (Fig. S13). Then, 2,2′-azinobis (3-ethylben zothiazoline-6-sulfonic acid) (ABTS) was selected as a radical scavenger to verify the radical generation efficiency of nanobullets (Fig. S14). Generally, ABTS could capture free radicals and exhibit characterized absorbance at 500–950 nm. A mixture of HA-ACVA-AZ NBs and ABTS was first cultured at 50 °C. The ABTS^+•^ concentration increased with the extended duration of incubation (Fig. 2g), which was not detected for HA-PA-AZ NBs (Fig. 2h). Additionally, the linearity of the absorbance of ABTS^+•^ at 736 nm as a function of incubation time at various temperatures is observed in Fig. 2i. Excitingly, there were negligible radicals generated at body temperature (37 °C), suggesting the good stability of radical initiators in nanobullets. It is worth mentioning that HA-ACVA-AZ NBs-***2*** achieved the best radical generation ability among HA-ACVA-AZ NBs-***1–3*** (Fig. S15). Then, the photothermal-inducible radical generation was also evaluated (Fig. 2j, k). It was clear that radicals could be generated efficiently only in the presence of both ZPA and ACVA. The results indicated that the generation of free radicals was triggered by heating ZPA@HA-ACVA-AZ NBs with laser. To further characterize the type of radicals, the electron spin resonance (ESR) technique was applied and 5,5-dimethyl-1-pyrroline N-oxide (DMPO) was used as a spin trap. As shown in Fig. 2l, alkyl radicals produced by ZPA@HA-ACVA-AZ NBs + laser were detected from the sextet (*α*_*N*_ = 15.2 G, *α*_*β–H*_ = 23.6 G), which was not observed for ZPA@HA-ACVA-AZ NBs in dark or HA-ACVA-AZ NBs in light. It was also noticed that the nanobullets split obviously upon external stimuli. In comparison, ZPA@HA-PA-AZ NBs without thermal-sensitive azo bonds maintained their integrity in structure upon laser irradiation, observed in DLS measurement (Fig. S16) and TEM images (Fig. S17).

### Cellular Uptake of Nanobullets

**Fig. 3:**
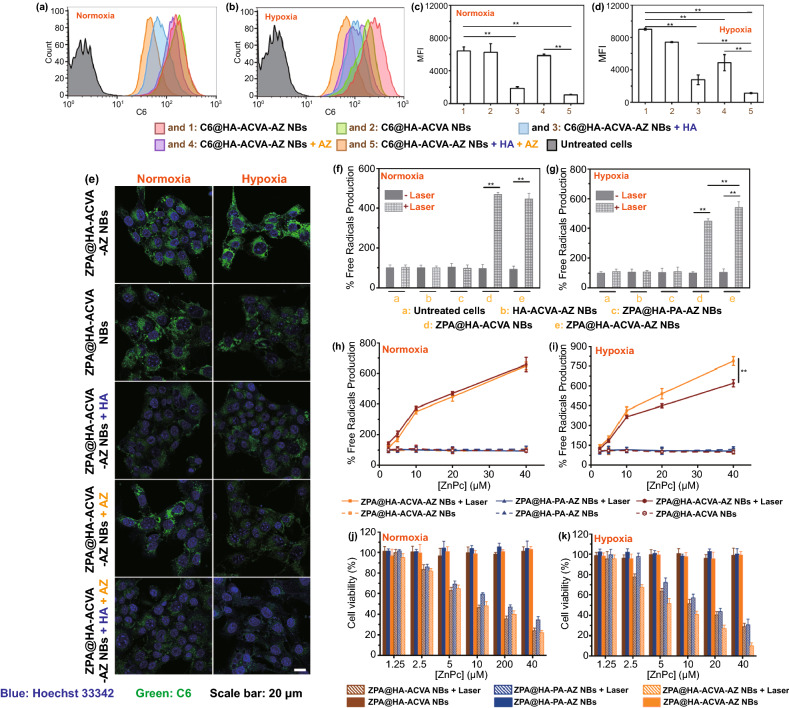
In vitro cellular uptake, alkyl free radical generation and therapeutics of ZPA@HA-ACVA-AZ NBs. Comparison of **a **and** c** fluorescence intensity profile and **b **and** d** relative intracellular fluorescence intensity of 4T1 cells in normoxic and hypoxic conditions after being incubated with C6@HA-ACVA NBs and C6@HA-ACVA-AZ NBs with or without pre-treatment of HA ([HA] = 10 mg/mL) and AZ ([AZ] = 20 μM)) for 24 h as determined by flow cytometry. **e** Fluorescence images of C6@HA-ACVA NBs and C6@HA-ACVA-AZ NBs with or without pre-treatment of HA ([HA] = 10 mg/mL) and AZ ([AZ] = 20 μM)) after 24-h incubation. The cells were stained with Hoechst 33,342 to visualize the nuclei (blue) and the fluorescence of C6 was shown in the color of green. Alkyl radical production induced by different samples in 4T1 cells in **f** normoxic and **g** hypoxic conditions with or without laser irradiation. Alkyl radical production induced by different ZPA-loaded NBs in 4T1 cells at various ZnPc concentrations in **h** normoxic and **i** hypoxic conditions with or without laser irradiation. MTT assay for measure the inhibited proliferation efficacy on 4T1 cells treated with different ZPA-loaded NBs with or without laser irradiation in **j** normoxic and **k** hypoxic conditions

As such, the ZPA@HA-ACVA-AZ NBs showed a great potential for PTT/TDT. The premise behind highly efficient synergistic PTT/TDT then was to internalize the nanobullets into hypoxic cancer cells. Since the expression of CA IX in hypoxic tumors up-regulates and CD44 is a cell surface receptor overexpressed on many malignant tumors [42, 43], the presence of HA (targeting CD44) and AZ (targeting CA IX) on the surface of nanobullets was anticipated to promote the cellular uptake of nanobullets into hypoxic tumor cells via the dual-targeting effect. To prove this, the cellular uptake of nanobullets was evaluated on 4T1 cells under normoxic (21% O_2_) and hypoxic (1% O_2_) conditions, respectively. Since the fluorescence emission of ZnPc in ZPA@HA-ACVA-AZ NBs was quenched, we prepared the coumarin 6 (C6)-encapsulated analogous systems, including C6@HA-ACVA NBs and C6@HA-ACVA-AZ NBs, for the cellular uptake study by using flow cytometry and confocal laser scanning microscopy (CLSM). Accordingly, more C6@HA-ACVA-AZ NBs were internalized into hypoxic 4T1 cells than C6@HA-ACVA NBs. Upon pre-treatment of cells by HA and AZ, the cellular uptake of C6@HA-ACVA-AZ NBs decreased significantly in hypoxic tumor cells due to the block of receptors on the cellular surface. As we expected, the influence of AZ on promoting cellular uptake was not much significant for normoxic cells with a low level of CA IX expression (Fig. 3a–d). Similar results were observed in CLSM visualization (Fig. 3e).

### Intracellular Free Radical Detection

Then, 2′,7′-dichlorodihydrofluorescein diacetate (DCFH-DA) was applied to evaluate the intracellular radical generation. According to the literature, free alkyl radical (R·) can convert to (RO·) to induce ROS formation in cells, which could react with DCFH without fluorescence signal to produce DCF with green fluorescence [44]. As shown in Fig. 3f, g, nanobullets containing ACVA components displayed efficient radical generation upon laser irradiation. More importantly, since the TDT was oxygen-independent, the cells treated with ZPA@HA-ACVA-AZ NBs + laser under hypoxia maintained the same level of alkyl radical generation as cells under normoxia. Furthermore, ZPA@HA-ACVA-AZ NBs with dual-targeting effect presented higher free radical production than ZPA@HA-ACVA NBs with single-targeting effect for hypoxic cells possibly attributed to the up-regulated expression of CA IX with high affinity to AZ [27, 45, 46]. Besides, the radical generation from nanobullets was in a concentration-dependent behavior (Fig. 3h, i). Compared with the traditional PDT, a ROS-mediated therapeutic modality [47], the main advantage of TDT is that the cytotoxic alkyl radicals also can be generated under the hypoxic tumor microenvironment. To prove it, we also detected the in vitro ROS production during ZnPc-mediated PDT under both hypoxic and normoxic conditions. As shown in Fig. S18a, the ROS generation efficiency in 4T1 cells upon 635-nm laser irradiation under hypoxia condition decreased significantly when compared with normoxic 4T1 cells.

### In vitro Cytotoxicity of ZPA@HA-ACVA-AZ NBs

We then examined the therapeutic efficacy in vitro through the MTT assay. In Figs. 3j, k and S17, all nanobullets showed low dark toxicity in 4T1 cells. However, upon laser irradiation, an obvious therapeutic effect could be obtained. ZPA@HA-PA-AZ NBs in light (single PTT) exhibited moderate cytotoxicity. After combining the PTT with TDT, the therapeutic efficacy was enhanced significantly. For example, for hypoxic 4T1 cells at the ZnPc concentration of 20 μM, the cell viability was 43% for the group of ZPA@HA-PA-AZ NBs + laser (single PTT), which decreased to 26% for ZPA@HA-ACVA-AZ NBs + laser (combined PTT/TDT). According to the previous studies, during the process of TDT, the produced free radicals are toxic to cancer cells. For the cells with adequate oxygen, the alkyl radicals could immediately oxidize cellular elements or interact with oxygen to produce secondary toxic substances (e.g., alkoxyl and peroxyl radicals). For the hypoxic tumor cells, the radicals have been proved to enhance intracellular lipid hydroperoxides, aggravate the damage of DNA and further trigger the apoptosis of tumor cells [14, 44, 48]. More importantly, attributed to enhanced cellular uptake of nanobullets, ZPA@HA-ACVA-AZ NBs displayed a 14% drop in cell viability as compared to ZPA@HA-ACVA NBs, which was not observed for cells incubated in normoxic condition. Because of the up-regulated CA IX expression for 4T1 cells in hypoxic condition, the CD44/CA IX dual-targeting effect of ZPA@HA-ACVA-AZ NBs played a crucial role in promoting the cellular uptake of nanobullets into hypoxic 4T1 cells (Fig. 3d, e) and further enhanced the therapeutic efficacy of NB-mediated synergistic PTT/TDT. These findings validated that the cytotoxicity of ZPA@HA-ACVA-AZ NBs was not affected by the oxygen level in cells and the special character of hypoxic tumor cells (high expression of CA IX) plays an important role in further enhancing the therapeutic efficacy. It is also worth mentioning that the ZnPc-mediated PDT efficacy against 4T1 cells under hypoxic condition decreased significantly compared with normoxic 4T1 cells (Fig. S18b). However, the ZPA@HA-ACVA-AZ NB-mediated PTT/TDT showed similar therapeutic efficacy for both normoxic and hypoxic 4T1 cells (Fig. 3j, k), further indicating the advantages of PTT/TDT against hypoxic tumors.

### In vivo Antitumor Activities of Nanobullets

**Fig. 4:**
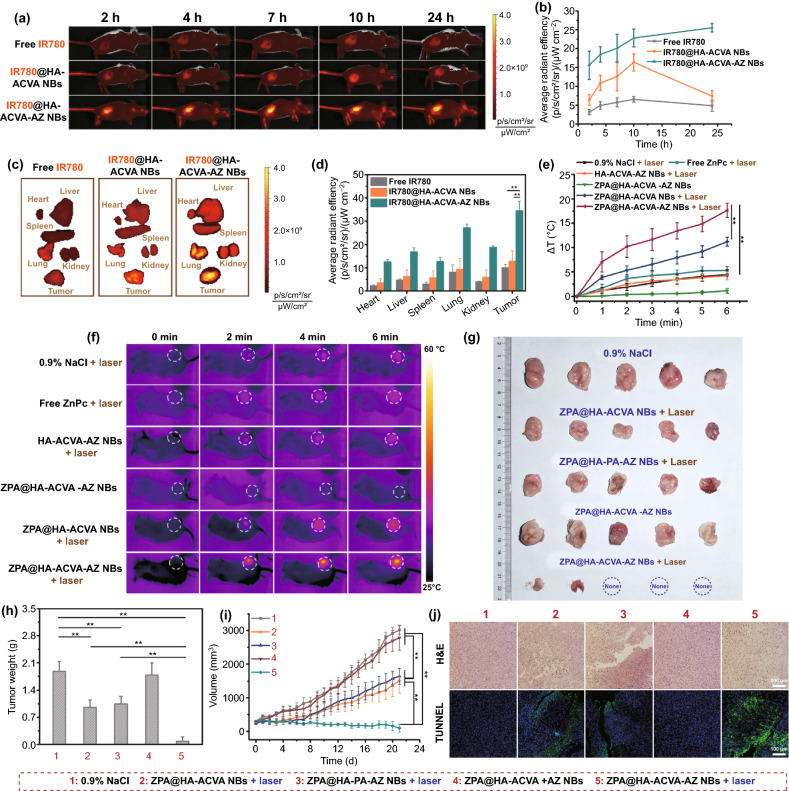
Biodistribution, in vivo photothermal performance and tumor inhibition ability of ZPA@HA-ACVA-AZ NBs. **a** NIR fluorescence imaging of balb/c mice bearing subcutaneous 4T1 tumors intravenously administrated with IR780@HA-ACVA-AZ NBs, IR780@HA-ACVA NBs and free IR780 at 2 h, 4 h, 7 h, 10 h and 24 h. **b** Average fluorescence intensity of IR780 in the tumor over time was measured with an in vivo imaging system. **c** Ex vivo NIR fluorescence imaging of IR780 fluorescence intensity in the harvested organs and tumors at 24 h post-administration (*n* = 4). **d** Quantitative analysis of IR780 fluorescence intensity in the harvested organs and tumors at 24 h post-administration. **e** Temperature change and **f** infrared thermal imaging at the tumor site of mice bearing 4T1 tumors treated with different drug formulations via the tail vein upon 808-nm laser irradiation (1 W cm^−2^, 10 min) (*n* = 4). **g** Morphology and **h** weight of tumors at the 21 days post-injection of 0.9% NaCl, ZPA@HA-ACVA NBs, ZPA@HA-PA-AZ NBs and ZPA@HA-ACVA-AZ NBs with or without laser exposure (1 W cm^−2^, 5 min). **i** Tumor growth profiles from each group (*n* = 5). **j** H&E and TUNEL stained images of the tumors from mice treated with various drug formulations on day 7 (*n* = 5)

In vivo biodistribution and antitumor efficacy of ZPA@HA-ACVA-AZ NBs were studied by using the 4T1 subcutaneous tumor xenograft model. Also, we prepared the IR780-encapsulated analogous nanobullets to endow the nanobullets with NIR fluorescence for in vivo imaging. As shown in Fig. 4a, b, after intravenous (*i.v.*) injection, the nanobullets could accumulate in tumor with the gradually increased IR780 fluorescence signal, which was much higher than free IR780. Additionally, dual-targeted IR780@HA-ACVA-AZ NBs presented higher fluorescence intensity than single-targeted IR780@HA-ACVA NBs, verifying the effect of AZ modification on facilitating tumor accumulation of nanobullets. From ex vivo fluorescence images of the excised organs at 24 h post-injection (Fig. 4c, d), the IR780@HA-ACVA-AZ NBs preferred to accumulate in tumors. Besides, the fluorescence from IR780@HA-ACVA-AZ NBs in tumors was 3.4-fold and 2.7-fold higher than free IR780 and IR780@HA-ACVA NBs, respectively. To further explore the in vivo behavior of ZPA-loaded nanobullets, the pharmacokinetic measurements of ZPA@HA-ACVA-AZ NBs were performed and compared with free ZnPc. As shown in Fig. S20. the ZnPc encapsulated in NBs exhibited a prolonged in vivo circulation time when compared with free ZnPc. For example, at 6 h post-injection, the ZnPc concentration in plasma in NBs group was 3.07 ug mL^−1^; however, ZnPc in free formulation group is only 0.3 μg mL^−1^. Furthermore, at end of the experiment, we can also detect the fluorescence signals of ZnPc in plasma in NBs group.

To detect the in vivo photothermal effect of nanobullets, the tumors were irradiated with 808-nm laser at 24 h post-injection of samples and the tumor temperature was monitored by an infrared thermal camera. As shown in Fig. 4e, f, ZPA@HA-ACVA NBs and ZPA@HA-ACVA-AZ NBs exhibited an obvious photothermal effect upon laser irradiation with temperature increment of 11 and 18 °C, respectively.

The tumor ablation ability of nanobullets was further examined. As displayed in Fig. 4g–j, a single administration of ZPA@HA-ACVA-AZ NBs combined with laser irradiation presented the optimal tumor ablation. On day 21 post-injection, there were 3 (out of 5) mice surviving with tumors disappeared. The tumor size and weight decreased to 96 mm^3^ and 0.08 g for the mice bearing remanent tumors. In comparison, the single PTT group (ZPA@HA-PA-AZ NBs + laser) displayed obviously enhanced tumor volume of 1636 mm^3^ and weight of 1.046 g. Similar results were also observed for the group with nanobullets of single-targeting effect (ZPA@HA-ACVA NBs + laser). It was suggested that ZPA@HA-ACVA-AZ NBs could not only efficiently target the CA IX overexpressed hypoxic tumor cells by the dual-targeting effect, but also generate alkyl radicals along with PTT for tumor therapy. Subsequently, hematoxylin and eosin (H&E) staining of tumor slices treated with ZPA@HA-ACVA-AZ NBs + laser presented more severe damage (Fig. 4j). These results agreed with the terminal deoxynucleotidyl transferase dUTP nick end labeling (TUNEL) staining assay, which further demonstrated the excellent synergistic therapeutic efficacy of ZPA@HA-ACVA-AZ NBs (Fig. 4j).

### Antilung Metastasis of Breast Cancer

**Fig. 5:**
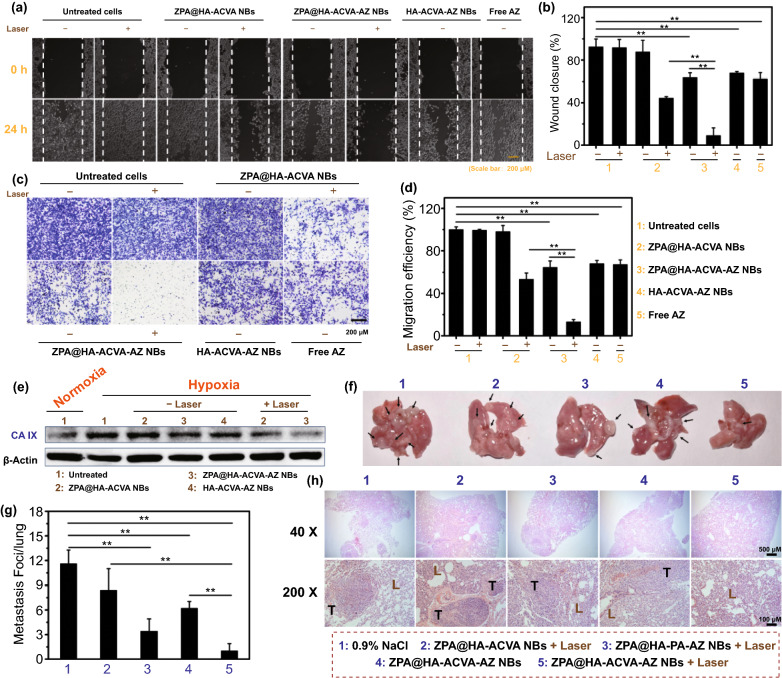
Lung metastasis inhibition from breast cancer by ZPA@HA-ACVA-AZ NBs. **a** Wound-healing ability, **b** wound closure efficiency, **c** transwell capacity, **d** transwell migration efficiency and **e** CA IX expression of 4T1 cells treated with varying drug formulations with or without 808-nm laser irradiation (1 W/cm^2^, 10 min). **f** Morphology, **g** metastasis foci and **h** H&E images of lungs from mice bearing 4T1 tumor injected with varying drug formulations with or without laser irradiation at a wavelength of 808 nm (1 W cm^−2^, 5 min) (n = 5). Black arrows in **f** represent the foci of tumor metastases. T and L in **h** represent the tumor and lung, respectively

It has been reported that CA IX inhibitors could inhibit the endogenous expression and catalytic activity of CA IX, thereby suppressing the metastases of hypoxic tumors. Furthermore, the inhibitory effect on cell migration by synergistic PTT/TDT is also required. The migration capacity of 4T1 cells after nanobullet-mediated PTT/TDT was evaluated using the wound-healing assay and transwell assay. In Fig. 5a, b, the nanobullets with AZ could moderately inhibit the recovery of the scratch wound. Further combining CA IX inhibition with synergistic PTT/TDT, achieved by ZPA@HA-ACVA-AZ NBs + laser, suppressed the scratch wound healing more effectively, resulting in a wound closure of only 9.04%. In the transwell assay, ZPA@HA-ACVA-AZ NBs + laser also displayed the strongest ability of inhibiting cell migration (Fig. 5c, d). From the Western blot results in Figs. 5e and S21, both HA-ACVA-AZ NBs and ZPA@HA-ACVA-AZ NBs with AZ could inhibit the expression of CA IX slightly and ZPA@HA-ACVA-AZ NBs + laser induced a more significant decrease of CA IX expression (58.4%), suggesting that suppressing migration of hypoxic 4T1 cells was highly relevant to the down-regulating the expression of CA IX. For in vivo study, lung metastasis is commonly diagnosed at the later stage of breast cancer, which is closely relevant to death in breast cancer [49–51]. After the tumor inhibition experiment, the lung-metastasized foci in each group were counted (Fig. 5f, g). Compared with the number of metastasized nodules of 12 for 0.9% NaCl, ZPA@HA-PA-AZ NBs + laser (PTT and CA IX inhibition) and ZPA@HA-ACVA-AZ NBs in dark (CA IX inhibition) displayed a declined number of nodules to 3 and 6, respectively. Fortunately, ZPA@HA-ACVA-AZ NBs + laser could almost suppress the lung metastasis of breast cancer cells with metastasized nodules of 1. The H&E staining for lungs of group treated with ZPA@HA-ACVA-AZ NBs + laser exhibited less detectable foci (Fig. 5h). Thus, it was demonstrated that a combination of CA IX inhibition and synergistic PTT/TDT could efficiently suppress lung metastasis of breast cancer cells.

### Evaluation of the Biosafety of Nanobullets

Biosafety is always a critical concern for nanomedicines in clinical applications [52]. First, the good hemocompatibility had been validated for blank HA-ACVA-AZ NBs with negligible hemolysis activity (< 6%) and ZPA@HA-ACVA-AZ NBs without erythrocyte agglutination (Fig. S22). Then, there had been no remarkable changes in body weight for the mice received PTT/TDT (Fig. S23a). The H&E images (Fig. S23b) also indicated no severe tissue damage in major organs after the *i.v.* injection of ZPA@HA-ACVA-AZ NBs plus laser, and the results of serum biochemistry suggested that this treatment succeeded in maintaining the level of AST, ALT, BUN and CRE in reference range with no hepatic or renal dysfunction. Besides, the results of blood routine analysis presented that all the measured indicators were in the normal range for the ZPA@HA-ACVA-AZ NBs plus laser irradiation group (Fig. S24).

## Conclusions

In conclusion, all-organic nanobullets were designed for the “precise strike” of hypoxic tumors through the dual-targeting effects from surface-modified HA and hypoxia-dependent factor CA IX inhibitor AZ and the synergistic PTT/TDT. With the property of hydrophobic and thermal-cleavable, the alkyl chain-functionalized initiator ACVA-HDA could be applied both as a hydrophobic segment of HA-based lipoid for nanobullet formation and free radical source for oxygen-independent TDT with the assistance of promising organic ZPA in the core of nanobullets as an efficient heat source. This nanosystem could not only generate heat for PTT but also produce photothermal-inducible alkyl radicals for TDT. Besides, the selection of HA as the “shell” of nanobullets which was further modified by hypoxia-dependent factor CA IX inhibitor AZ promoted the accumulation of nanobullets in hypoxic tumors by a CD44/CA IX dual-targeting effect. More importantly, the combination of CA IX inhibition by AZ and synergistic PTT/TDT possessed incomparable therapeutic advantages over traditional (ROS-mediated) cancer treatment in suppressing the growth of both hypoxic tumors and metastases hypoxic tumors. Therefore, our smart nanobullets have great potential to conquer the pitfalls of hypoxic tumors and diseases for effective synergistic treatments.

## Supplementary Information

Below is the link to the electronic supplementary material.

Supplementary file1 (DOCX 24375 KB)
