# Supplementary material for: Targeting Hypoxic Tumors with Hybrid Nanobullets for Oxygen-Independent Synergistic Photothermal and Thermodynamic Therapy
Source: Nanomicro Lett. 2021 Mar 31;13:99. doi: 10.1007/s40820-021-00616-4 (PMC8012440; doi:10.1007/s40820-021-00616-4)
Supplement: Supplementary file 1 — Supplementary file1 (DOCX 24375 KB) [file 40820_2021_616_MOESM1_ESM.docx]

Nano-Micro Letters

Supporting Information for

**Targeting Hypoxic Tumors with Hybrid Nanobullets for Oxygen-Independent Synergistic Photothermal and Thermodynamic Therapy**

Di Gao^1, †^, Ting Chen^1, †^, Shuojia Chen^1^, Xuechun Ren^1^, Yulong Han^2, 3^, Yiwei Li^2, 3^, Ying Wang^1^, Xiaoqing Guo^1^, Hao Wang^1^, Xing Chen^4^, Ming Guo^3^, Yu Shrike Zhang^5^, Guosong Hong^6^, Xingcai Zhang^2,3,^ *, Zhongmin Tian^1,^ *, Zhe Yang^1,^ *

^1^The Key Laboratory of Biomedical Information Engineering of Ministry of Education, School of Life Science and Technology, Xi’an Jiaotong University, Xi’an 710049, P. R. China

^2^John A. Paulson School of Engineering and Applied Sciences, Harvard University, Cambridge, Massachusetts 02138, United States

^3^School of Engineering, Massachusetts Institute of Technology, Cambridge, MA, 02139, United States

^4^School of Public Health, Guangxi Medical University, Nanning 530000, P. R. China

^5^Division of Engineering in Medicine, Department of Medicine, Brigham and Women's Hospital, Harvard Medical School, Cambridge, MA, 02139, United States

^6^Department of Materials Science and Engineering, Stanford University, Stanford, CA 94305, United States

† Di Gao and Ting Chen contributed equally to this work

*Corresponding author. E-mail: yangzhe@xjtu.edu.cn (Zhe Yang) zmtian@mail.xjtu.edu.cn (Zhongmin Tian) or [xingcai@mit.edu](mailto:xingcai@mit.edu) (Xingcai Zhang)

Nano-Micro Letters

**Supplementary Tables and Figures**

**
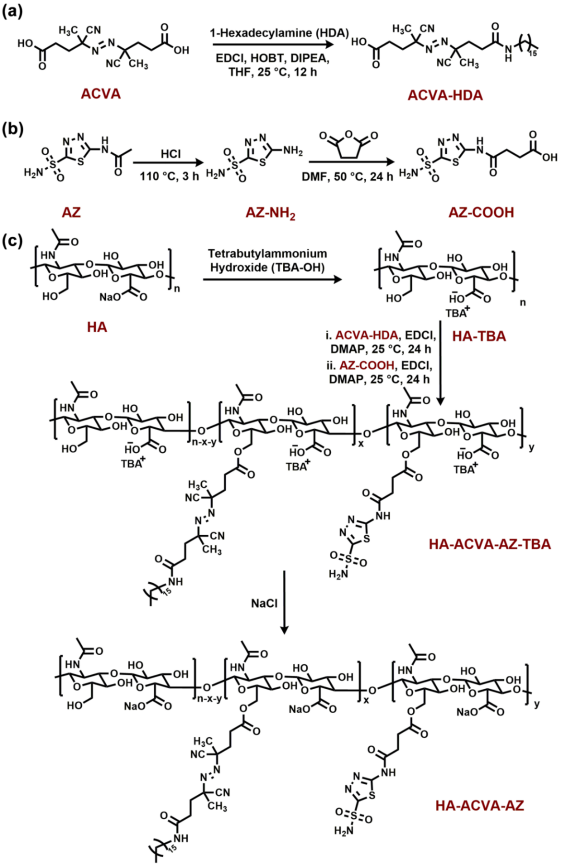
**

**Fig. S1** **a** Synthesis of ACVA-HDA. **b** Synthesis of AZ-COOH. **c** Synthetic route of the amphiphilic polymer HA-ACVA-AZ

Nano-Micro Letters


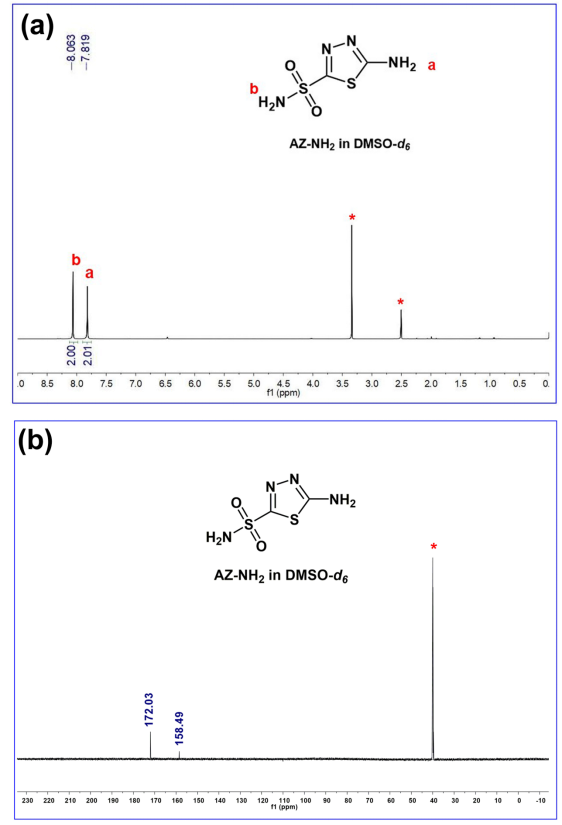


**Fig. S2** **a** ^1^H NMR and **b** ^13^C {^1^H} NMR spectrum of AZ-NH_2_ in DMSO-*d_6_*

Nano-Micro Letters

**
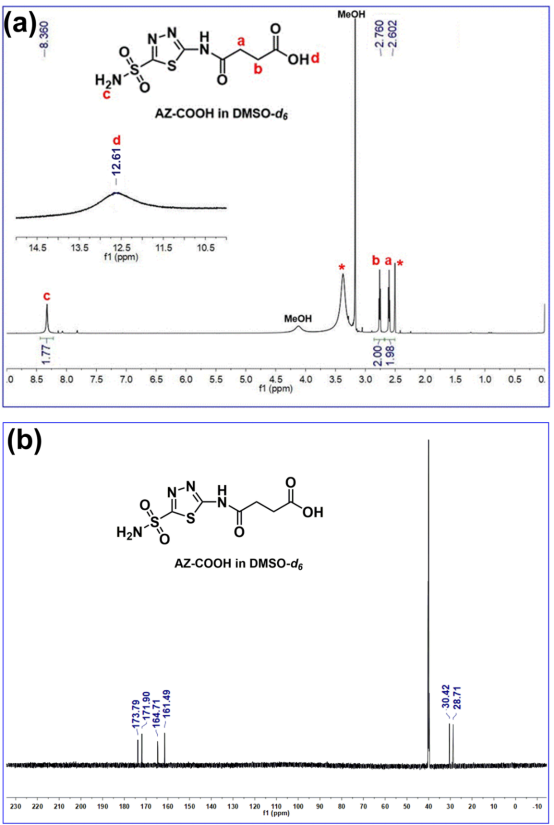
**

**Fig. S3** **a** ^1^H NMR and **b** ^13^C {^1^H} NMR spectrum of AZ-COOH in DMSO-*d_6_*

Nano-Micro Letters

**
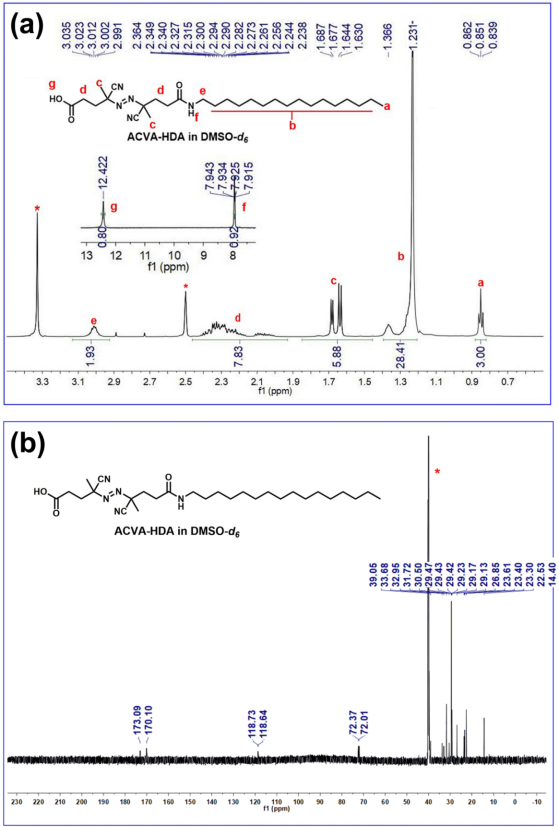
**

**Fig. S4** **a** ^1^H NMR and **b** ^13^C {^1^H} NMR spectrum of ACVA-HDA in DMSO-*d_6_*

Nano-Micro Letters


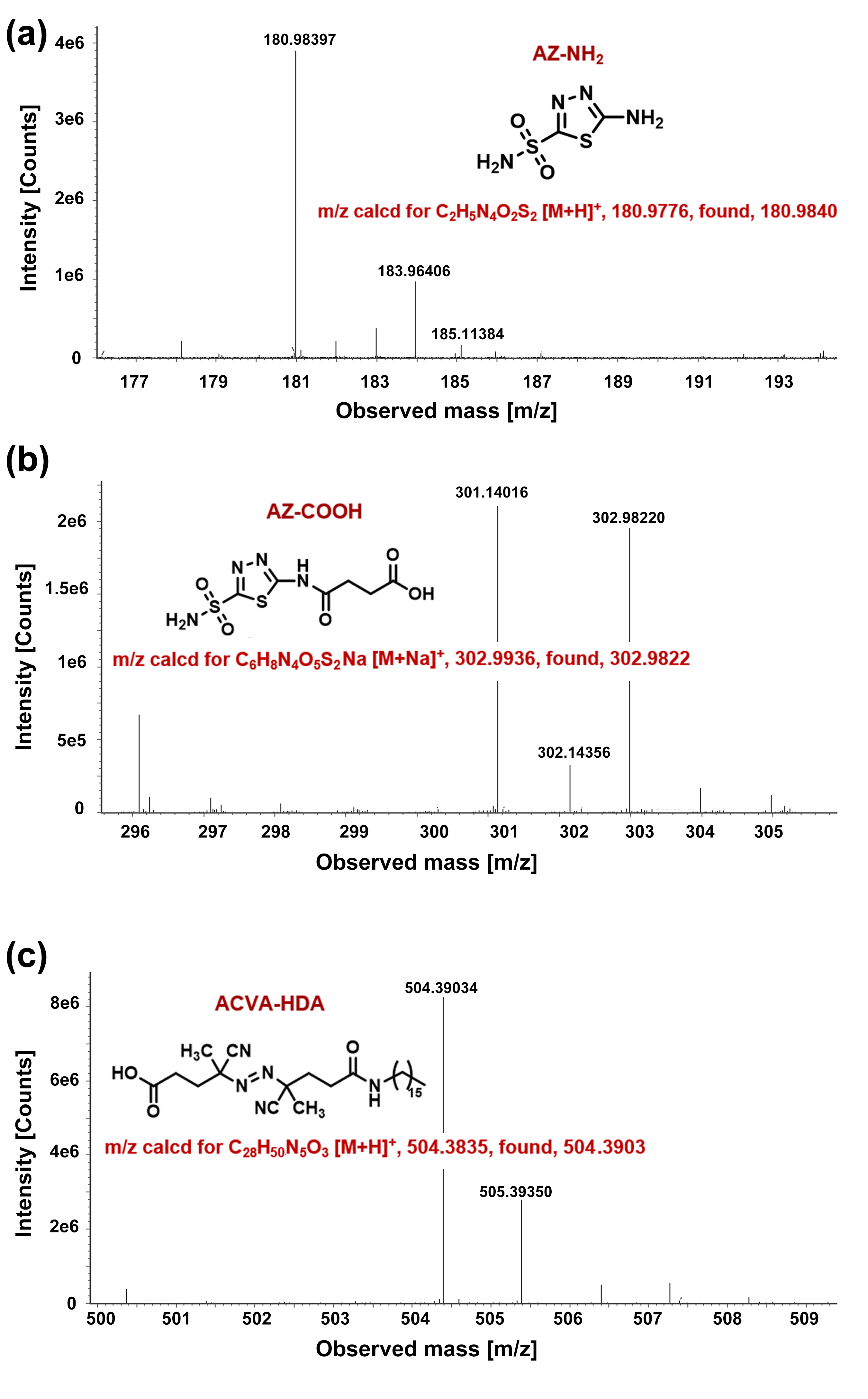


**Fig. S5** ESI mass spectrum: **a** AZ-NH_2_, **b** AZ-COOH, **c** ACVA-HDA


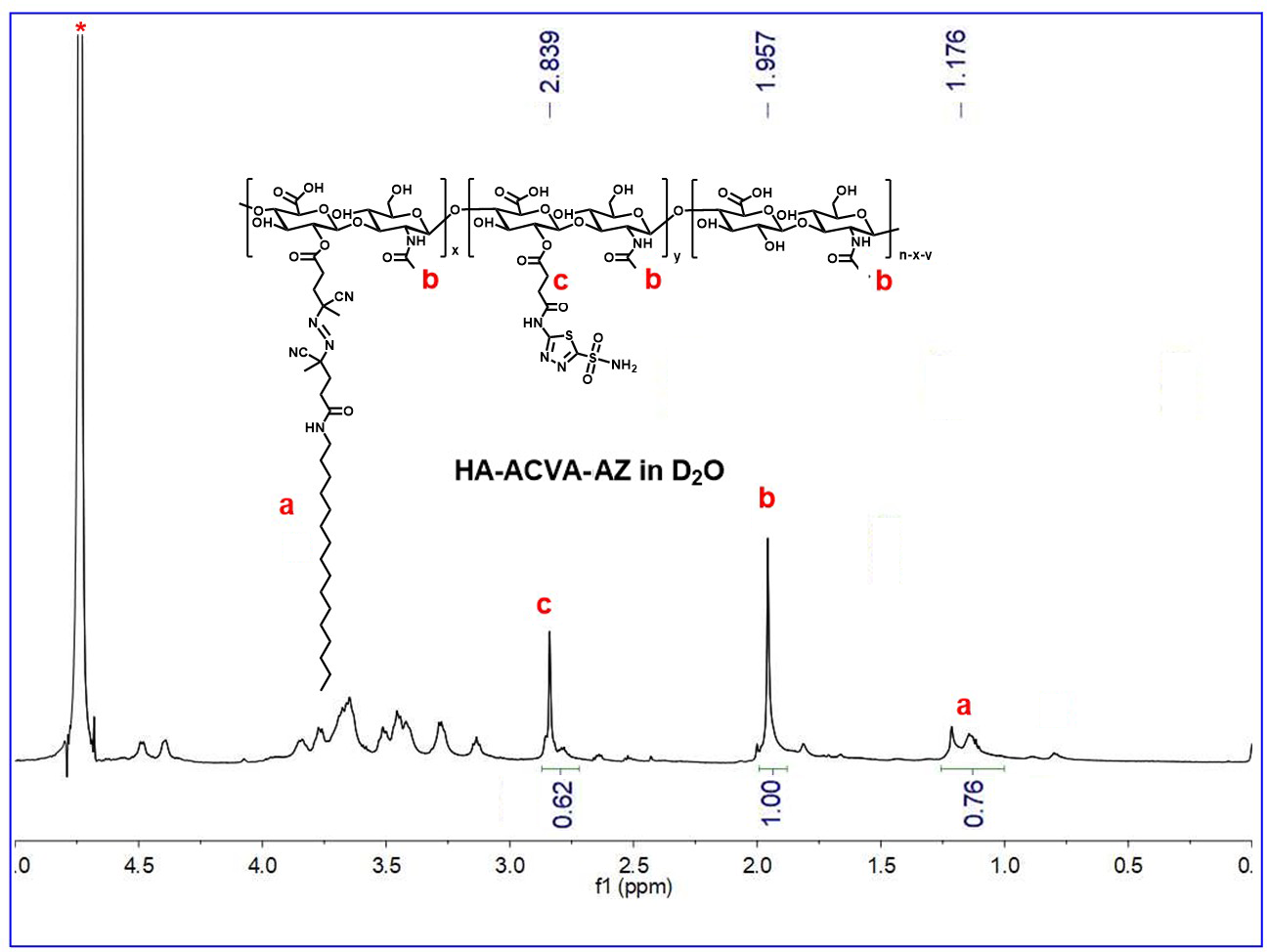
Nano-Micro Letters



**Fig. S6** ^1^H NMR spectrum of HA-ACVA-AZ in D_2_O

**Fig. S7** ^1^H NMR spectrum of HA-PA-AZ in D_2_O

Nano-Micro Letters


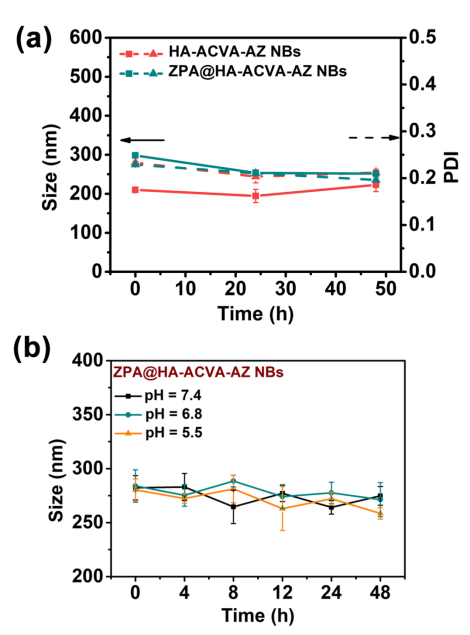

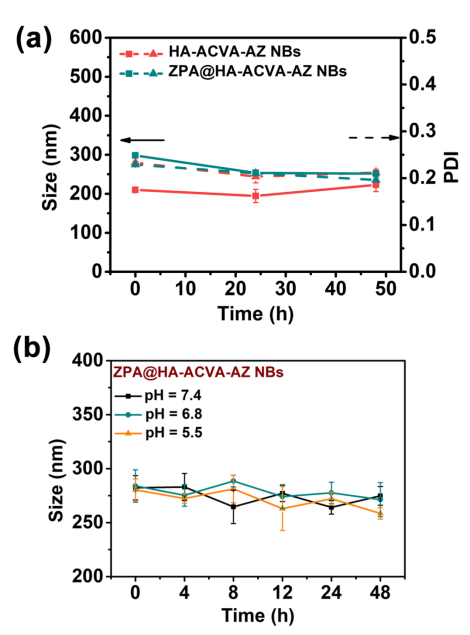


**Fig. S8** **a** Changes of HA-ACVA-AZ NBs’ and ZPA@HA-ACVA-AZ NBs’ size and PDI in PBS containing FBS (10%, v/v) at 37 °C for 48 h. **b** Changes of HA-ACVA-AZ NBs’ and ZPA@HA-ACVA-AZ NBs’ size in different PBS (pH 7.4, 6.8 and 5.5) at 37 °C for 48 h. Data are shown as the mean ± standard deviation (n = 3)


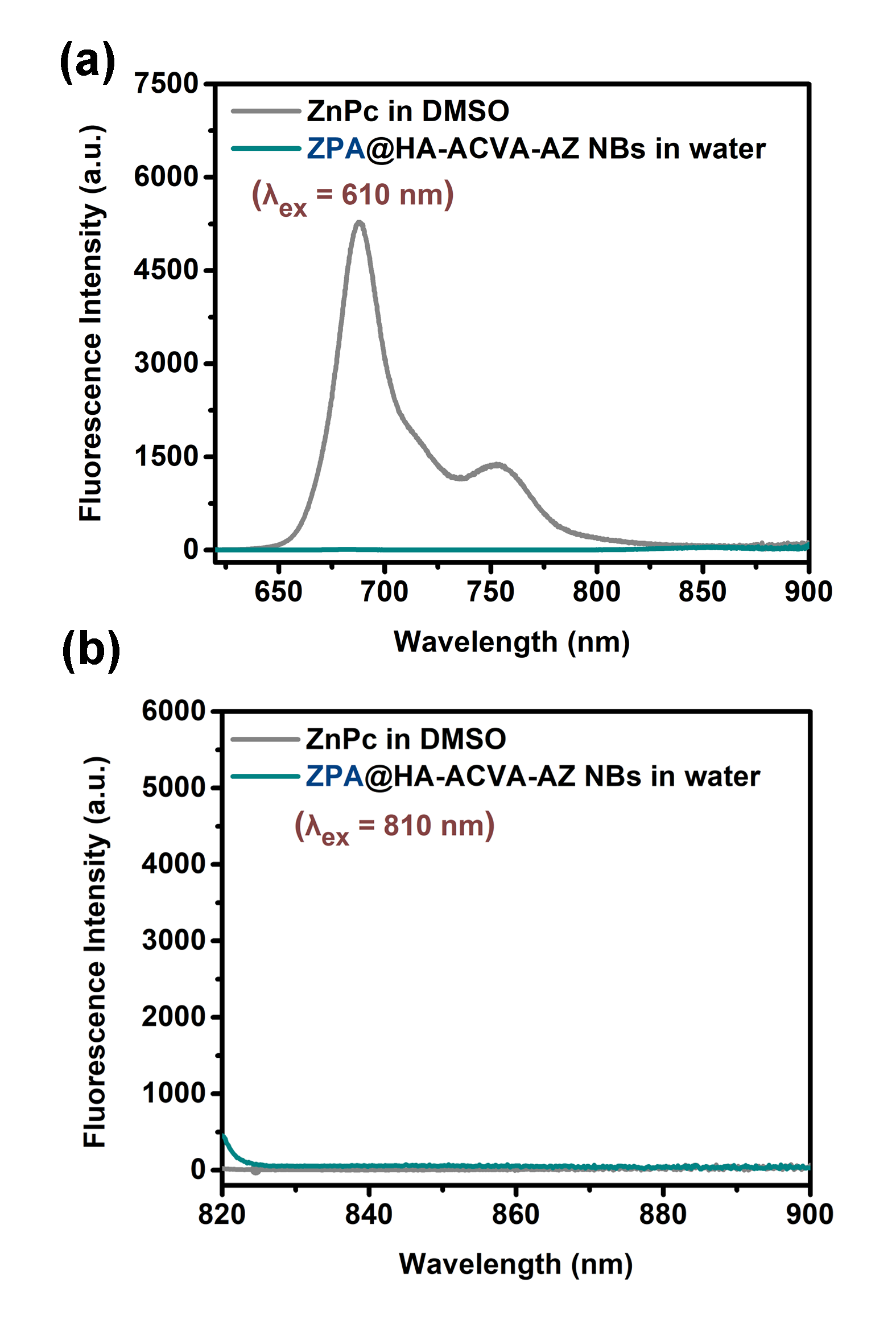


**Fig. S9** Fluorescence spectra of ZPA@HA-ACVA-AZ NBs in deionized water and ZnPc in DMSO with excitation wavelength at **a** 610 nm and **b** 810 nm ([ZnPc] = 10 μM)

Nano-Micro Letters


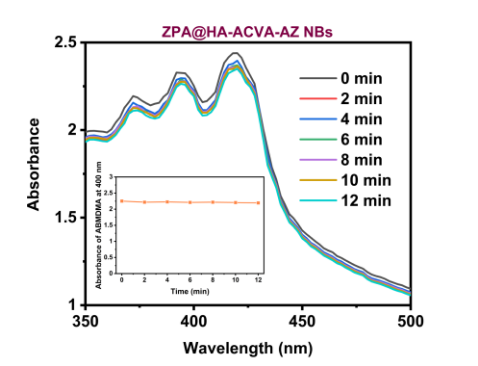


**Fig. S10** Change in absorption spectrum of ABMDMA in deionized water in the presence of ZPA@HA-ACVA-AZ NBs upon laser irradiation (808 nm, 1 W cm^-2^, [ZnPc] = 5 μM). The inset shows the variation of absorbance at 400 nm with time

**
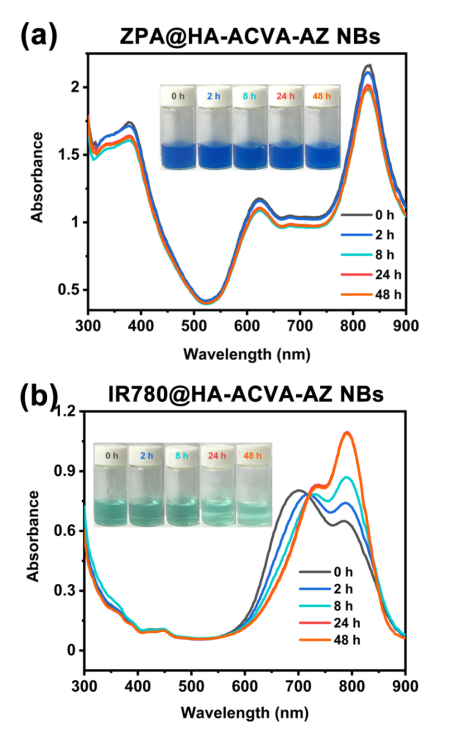
**

**Fig. S11** Change in the absorption spectrum of **a** ZPA@HA-ACVA-AZ NBs and **b** IR780@HA-ACVA-AZ NBs in PBS (pH = 7.4) at 37 °C over time

Nano-Micro Letters


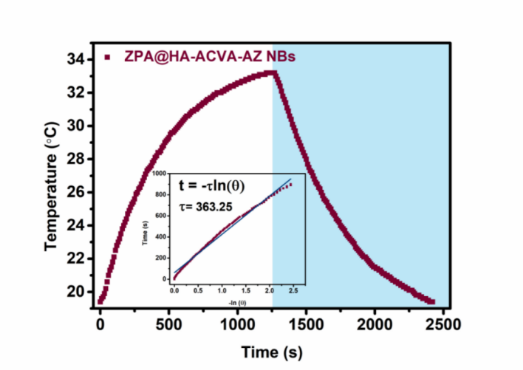


**Fig. S12** Photothermal profile of ZPA@ HA-ACVA-AZ NBs in deionized water irradiated by 808 nm NIR laser ([ZnPc] = 20 μM, 1 W/cm^2^) for 21 min, followed by natural cooling to room temperature. (Inset figure: determination of time constant for heat transfer (τ) of the system)

**
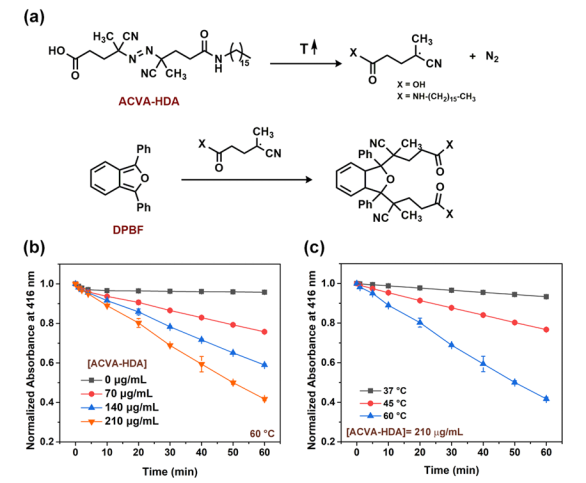
**

**Fig. S13** **a** The mechanism for the degradation of DPBF by alkyl radicals upon heating ACVA-HDA. **b** Degradation rate of DPBF sensitized by ACVA-HDA in DMF of different concentrations at 60 °C. **c** Degradation rate of DPBF sensitized by ACVA-HDA in DMF under various temperatures at concentration of 210 μg/mL


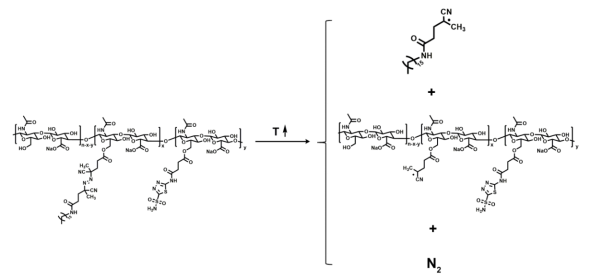
Nano-Micro Letters

**Fig. S14** Illustration of the principle and process of free radical generation upon heat treatment of HA-ACVA-AZ


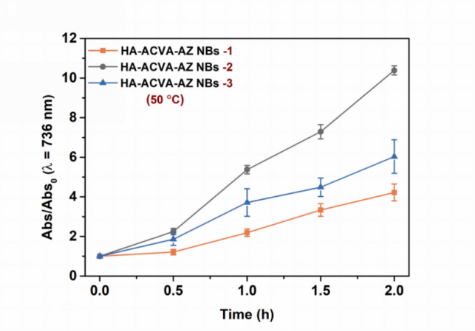


**Fig. S15** Generation of ABTS^+•^ as induced by the free radicals released from different blank HA-ACVA-AZ NBs at 50 °C ([NBs] = 5 mg/mL)

Nano-Micro Letters


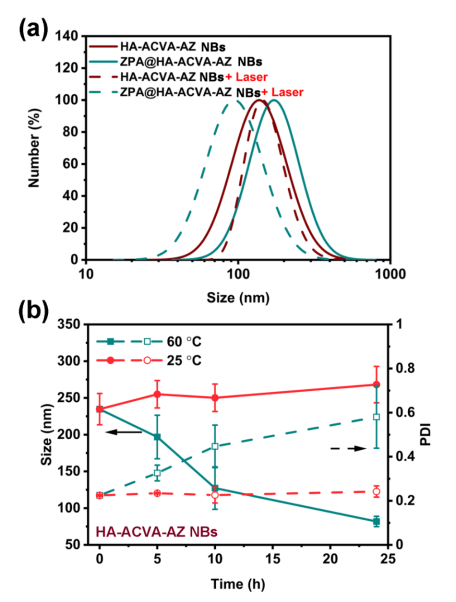

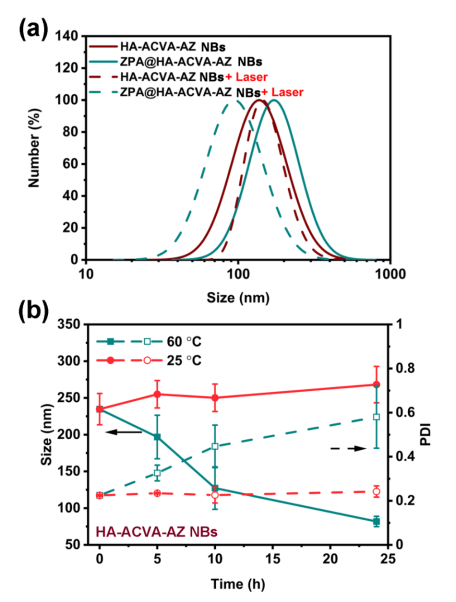


**Fig. S16** **a** Particle size distribution of blank HA-ACVA-AZ NBs and ZPA@HA-ACVA-AZ NBs with or without laser irradiation (808 nm, 1 W/cm^2^, 10 min). **b** Change in the size and PDI of blank HA-ACVA-AZ NBs at 25 °C and 60 °C over time


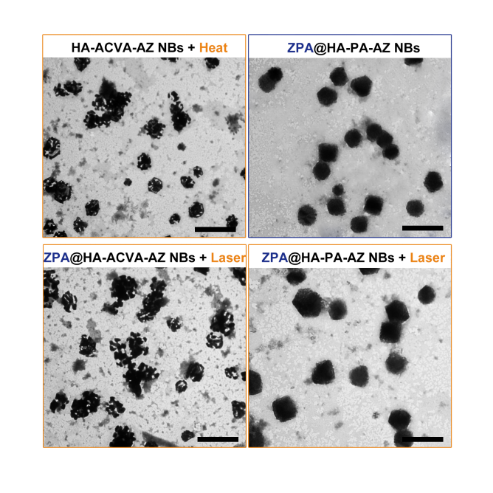


**Fig. S17** TEM images of blank HA-ACVA-AZ NBs upon heat treatment, ZPA@HA-ACVA-AZ NBs with laser irradiation and ZPA@HA-PA-AZ NBs with or without laser irradiation. (scale bar: 500 nm)

Nano-Micro Letters


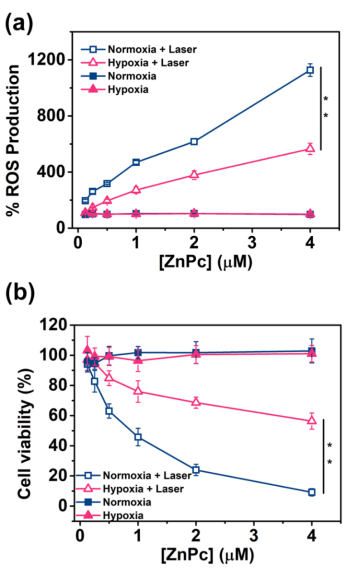

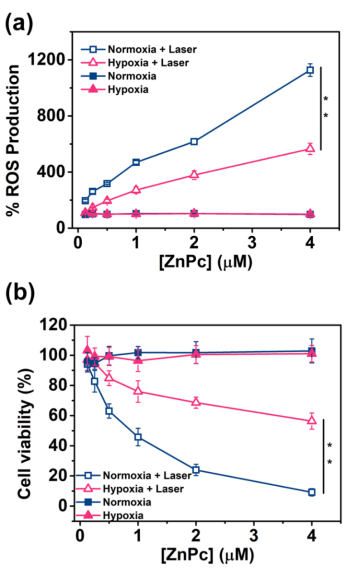


**Fig. S18 a** ROS production induced by ZnPc in 4T1 cells in the absence (closed symbols) or presence (open symbols) of 635 nm laser irradiation (30 mW/cm^2^, 5 min) under normoxia or hypoxia conditions. **b** Cytotoxicity induced by ZnPc on 4T1 cell in the absence (closed symbols) or presence (open symbols) of 635 nm laser irradiation (30 mW/cm^2^, 5 min) under normoxic or hypoxic conditions

**
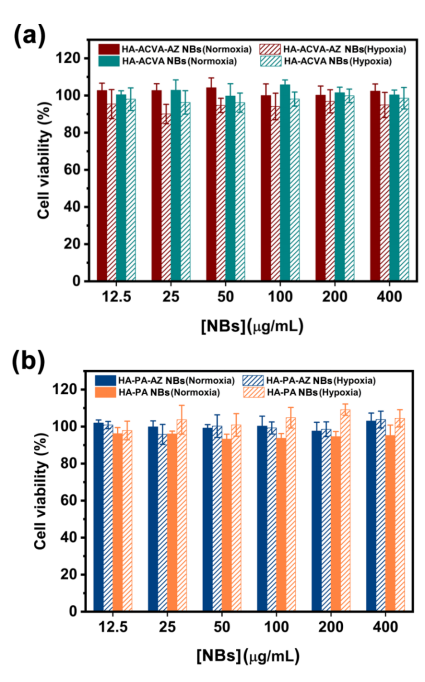

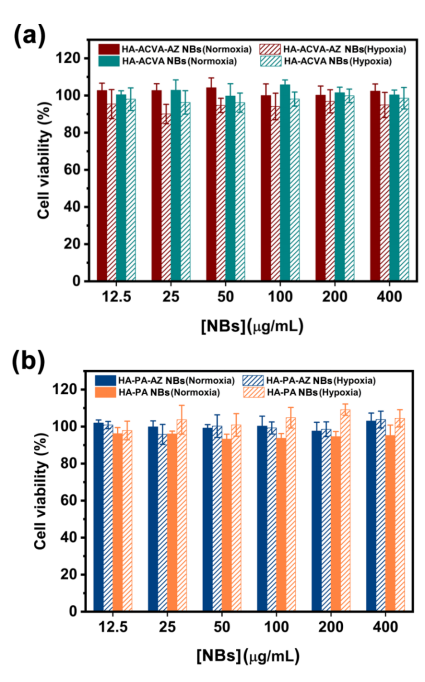
**

**Fig. S19** Cell viability of 4T1 cells after incubation with various concentrations of **a** HA-ACVA NBs and HA-ACVA-AZ NBs, **b** HA-PA NBs and HA-PA-AZ NBs for 24 h in normoxic and hypoxic conditions

Nano-Micro Letters


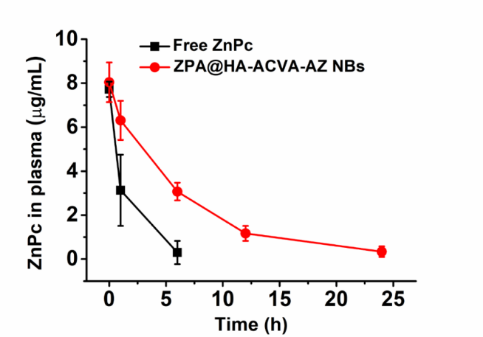


**Fig. S20** Plasma ZnPc concentration versus time after intravenous administration of Free ZnPc and ZPA@HA-ACVA-AZ NBs for 24 h at an equivalent dose of 2 mg ZnPc per kg of mice body (n=5)

**
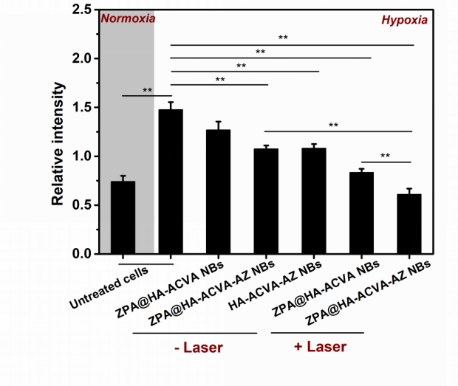
**

**Fig. S21** Quantitative analysis of the western blotting bands for CA IX expression of 4T1 cells treated with varying drug formulations with or without 808 nm laser irradiation (1 W/cm^2^, 10 min) by using ImageJ

Nano-Micro Letters


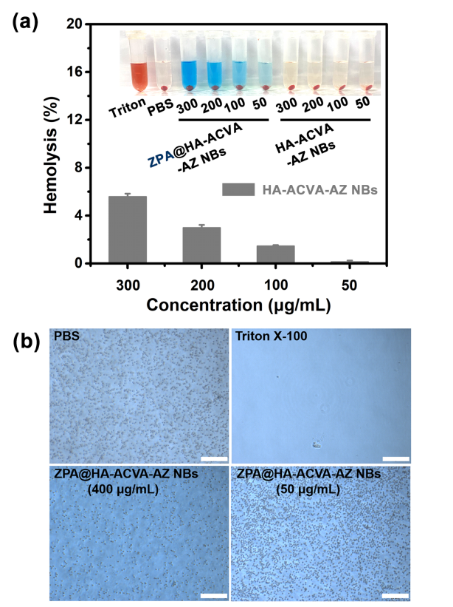

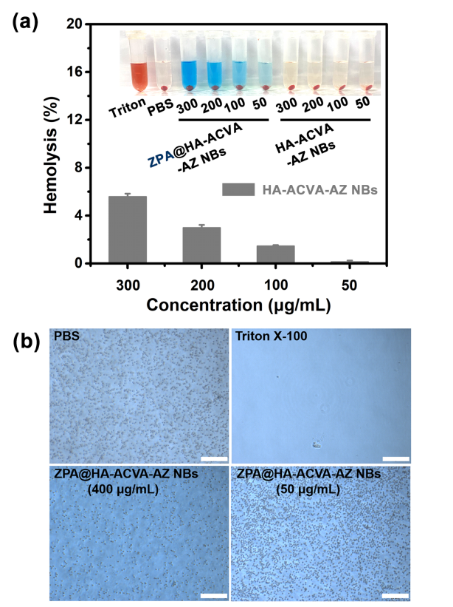


**Fig. S22** **a** Hemolytic activity of HA-ACVA-AZ NBs at different concentrations. The inset shows the images of erythrocyte incubated with HA-ACVA-AZ NBs and ZPA@HA-ACVA-AZ NBs after centrifugation. **b** Erythrocyte images after treatment of ZPA@HA-ACVA-AZ NBs at 50 μg/mL and 400 μg/mL concentration using a microscope (scale bars: 50 μm)


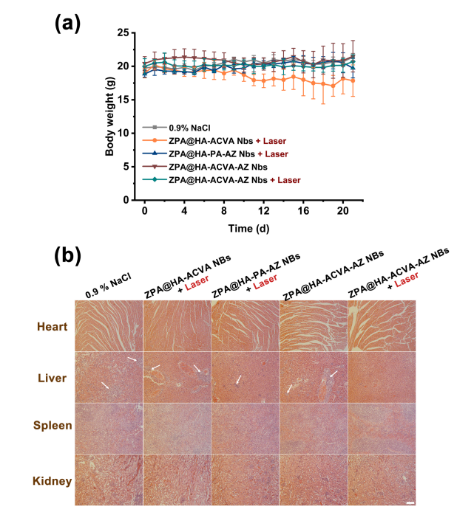


**Fig. S23 a** Body weight changes and **b** H&E images (on day 21) of normal tissues in balb/c mice bearing 4T1 tumor after systematic injection of different drug formulations (scale bars: 200 μm)

Nano-Micro Letters


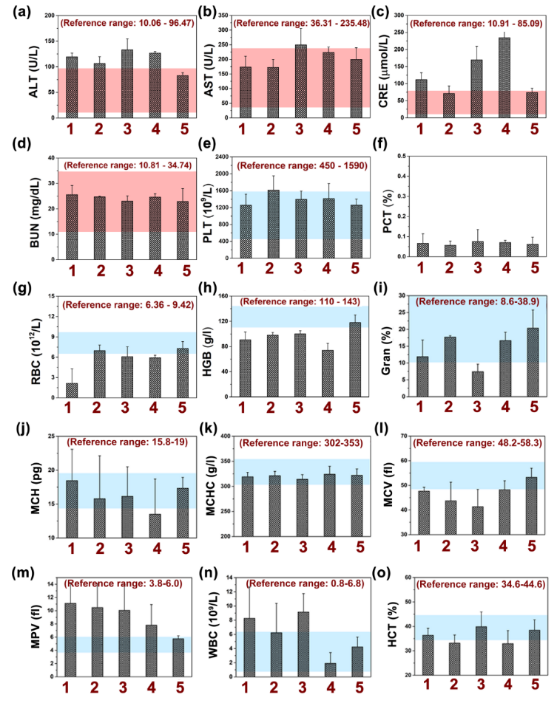


**Fig. S24** The blood level of **a** ALT, **b** AST, **c** CRE, **d** BUN in plasma and **e-o** the routine blood examination on day 21 of balb/c mice bearing 4T1 tumors after systematic injection of different drug formulations. (1: 0.9% NaCl, 2: ZPA@HA-ACVA NBs + laser, 3: ZPA@HA-PA-AZ NBs + laser, 4: ZPA@HA-ACVA-AZ NBs, 5: ZPA@HA-ACVA-AZ NBs + laser)

Nano-Micro Letters

**Table S1** Characterization of HA-based amphiphilic lipoid.

| **Polymers** | **Feed molar ratio^a^** | | **Calculated grafting degree^b^** | |
| --- | --- | --- | --- | --- |
|  | **ACVA-HDA or PA** | **AZ** | **ACVA-HDA or PA** | **AZ** |
| HA-ACVA-***1*** | 20% | - | 2.5% | - |
| HA-ACVA-AZ-***1*** | 20% | 30% | 3.2% | 19.5% |
| HA-ACVA -***2*** | 50% | - | 7.9% | - |
| HA-ACVA-AZ-***2*** | 50% | 30% | 8.1% | 18.8% |
| HA-ACVA-***3*** | 70% | - | 11.4% | - |
| HA-ACVA-AZ-***3*** | 70% | 30% | 11.9% | 15.0% |
| HA-PA | 50% | - | 8.3% | - |
| HA-PA-AZ | 50% | 30% | 8.1% | 17.9% |

^a^ Feed molar ratio of ACVA-HDA, PA and AZ to the hydroxyl groups of the side chain of HA during the synthesis of HA-based amphiphilic polymers.

^b^ Grafting degree of ACVA-HDA, PA and AZ on the side chain of HA calculated based on ^1^H NMR spectra.

**Table S2** Characterization of the blank HA-ACVA-AZ NBs and ZPA@HA-ACVA-AZ NBs prepared by different HA-based amphiphilic lipoids.

| **NBs** | **Size (nm)** | **PDI** | **Zeta potential (mV)** | **Entrapment efficiency %** | **Drug loading %** |
| --- | --- | --- | --- | --- | --- |
| HA-ACVA-AZ NBs-*1* | 251.74±30.97 | 0.103±0.01 | -29.03±2.57 | - | - |
| HA-ACVA-AZ NBs-*2* | 271.83±22.31 | 0.149±0.01 | -44.49±8.95 | - | - |
| HA-ACVA-AZ NBs-*3* | 241.73±32.17 | 0.325±0.01 | -23.46±1.02 | - | - |
| ZPA@HA-ACVA-AZ NBs-*1* | 360.27±26.99 | 0.157±0.04 | -30.04±3.12 | 73.06 % | 6.81 % |
| ZPA@HA-ACVA-AZ NBs*-2* | 297.99±21.89 | 0.126±0.03 | -34.70±6.23 | 89.65 % | 8.23 % |
| ZPA@HA-ACVA-AZ NBs*-3* | 286.49±12.94 | 0.283±0.02 | -25.64±5.49 | 72.86 % | 6.79 % |
